# TMS Motor Mapping Methodology and Reliability: A Structured Review

**DOI:** 10.3389/fnins.2021.709368

**Published:** 2021-08-19

**Authors:** Rachel E. Sondergaard, Davide Martino, Zelma H. T. Kiss, Elizabeth G. Condliffe

**Affiliations:** ^1^Department of Clinical Neuroscience, University of Calgary, Calgary, AB, Canada; ^2^Hotchkiss Brain Institute, University of Calgary, Calgary, AB, Canada; ^3^Department of Psychiatry, University of Calgary, Calgary, AB, Canada; ^4^Department of Pediatrics, University of Calgary, Calgary, AB, Canada

**Keywords:** transcranial magnetic stimulation, motor cortex, motor mapping, TMS methodology, neuronavigation, hotspot, centre of gravity, muscle mapping

## Abstract

Motor cortical representation can be probed non-invasively using a transcranial magnetic stimulation (TMS) technique known as motor mapping. The mapping technique can influence features of the maps because of several controllable elements. Here we review the literature on six key motor mapping parameters, as well as their influence on outcome measures and discuss factors impacting their selection. 132 of 1,587 distinct records were examined in detail and synthesized to form the basis of our review. A summary of mapping parameters, their impact on outcome measures and feasibility considerations are reported to support the design and interpretation of TMS mapping studies.

## Introduction

Motor mapping with transcranial magnetic stimulation (TMS) is a non-invasive technique used to probe motor cortical representation in humans. TMS mapping can evaluate features of motor representations and be used to draw conclusions about muscle group somatotopy within the motor cortex. TMS mapping can serve as a pre-surgical planning tool for tumor resection, a functional assessment tool following stroke or injury, a means of assessing development in children, and as a technique to probe basic questions relating to descending motor control. These applications demand rigorous methodologies, and careful interpretation of outputs; yet, efforts to standardize methodology remain preliminary (Krieg et al., 2017). Attempts to replicate experimental conditions are complicated by partial methodologies reported in literature (Cavaleri et al., 2017), perhaps contributing to the idea that TMS results are difficult to reproduce (Héroux et al., 2017), with large variability in response misinterpreted or obscured by statistical treatments (Héroux, 2019; Massé-Alarie et al., 2019).

Methodological sources of variability attributable to the use of TMS, and protocols to minimize the influence of this variability have been identified in prior reports (Chipchase et al., 2012; Krieg et al., 2017). Rigorous investigation has led to the recommendation that adaptive threshold testing be utilized to determine an individual’s resting motor threshold (RMT; Rossini et al., 2015), and that the minimum number of stimuli required to offset response variability within and between sessions is 5 and 10, respectively, (Cavaleri et al., 2017). Furthermore, a checklist has been developed listing aspects of motor TMS studies which should be reported and controlled (Chipchase et al., 2012). Notably, many of these methodological features have not been systematically assessed to determine optimal parameter selection for broad application to TMS.

In the motor mapping literature, variability also exists in mapping protocols, making it challenging to interpret whether differences in outcome measures across studies are attributable to methodological details or to differences in physiological processes. For instance, two studies published within a time span of several months both investigated the influence of navigation system use on outcome measures, with conflicting results (Julkunen et al., 2009; Jung et al., 2010). Similarly, separate studies have found reliability of area or volume of the map representing the same hand muscle to be low, moderate or high (Mortifee et al., 1994; Oliveri et al., 1999; Weiss et al., 2013).

There are several controllable elements of the mapping procedure that can influence outcome measures in these experiments, including: (1) use of a navigation system, (2) motor state of the individual during an experiment, (3) use of and spacing of a grid to produce the maps, (4) stimulator types and their positioning, (5) stimulator intensity at which the maps are produced, and (6) the selection of muscles to be mapped. Careful selection of the values of these parameters in defining motor mapping protocols seems necessary to minimize experimental sources of error. This would lead to improved quality, repeatability and ease of interpretation of study results. Given the lack of consensus on these methods, we examined the validity of the techniques reported in these studies. We also evaluated the practicality of implementing the methods reported in terms of participant comfort, test duration and equipment requirements. The aim of this structured review is to detail critical aspects of motor mapping methodology to aid in the design of robustly justified experiments with reproducible outcomes.

## Motor Mapping Parameter Selection and Evaluation

**FIGURE 1:**
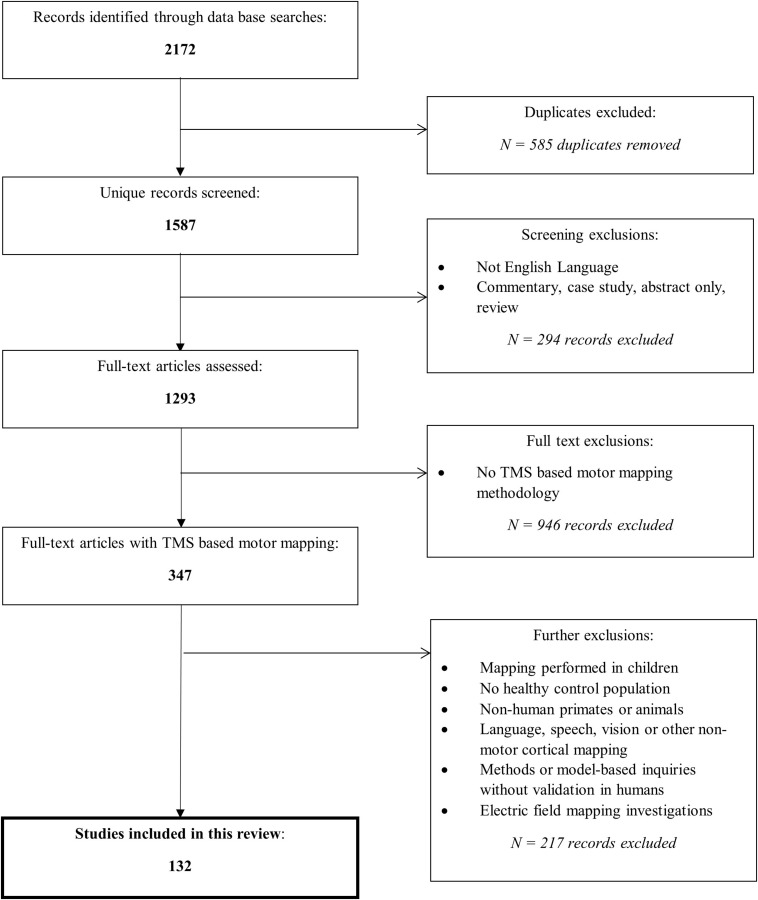
Overview of search process. The search strategy differed slightly between the databases due to their search convention, however, can be generally summarized by: (brain OR cortex OR cerebral) AND (map OR localization OR localization OR organization OR representation OR reorganization) AND (reprodu* OR repeat* OR valid* OR stab* OR verif* OR *re-test OR reliab* OR variab*) AND (transcranial magnetic stimulation). Reasons for exclusions of record screening included: case studies, poster abstracts, reviews, and commentaries or entries that were not written in English. Reasons for excluding full-text articles were: not studying TMS based motor mapping such as mapping of the visual system, evaluating motor response strength (MEPs) but not geographical map representation, or modeling studies without human experimental validation, mapping studies with different primary outcome measures (for example, latency) than those of interest in this review were also excluded. Additional exclusions are stated. Each database was queried using the criteria outlined on October 1st, 2020; the search included entries indexed on the databases at that time.

MEDLINE, EMBASE, Scopus, and Web of Science databases were utilized to perform a search focusing on studies evaluating a TMS method(s) to map motor pathways in humans. A summary of our search strategy including specific keywords for each engine and exclusion criteria is outlined in Figure 1.

We reviewed six distinct mapping parameters: navigation techniques, motor state, grid use, stimulator system factors, stimulus intensity, and muscle selection. We evaluated the effect of mapping parameter choices on the reliability of the mapping outcome measures, using intraclass correlation coefficients (ICCs) when reported. We also commented on the ease of use of these parameter choices. Box 1 defines these TMS motor mapping outcome measures, all of which require the measurement of motor-evoked potentials (MEP) using electromyography (EMG).

**Box 1. Mapping outcome measure definitions.**

**Hotspot (HS):** The hotspot is the stimulated site producing the largest MEP response. It may be decomposed into Cartesian reference using HS_x_ and HS_y_ in reference to the medial-lateral and antero-posterior axes, respectively.

**Centre of Gravity (CoG):** The center of gravity is the location on the grid most likely to produce the largest MEP when considering the size and location of all MEP responses. It may be decomposed into Cartesian reference using CoG_x_ and CoG_y_ in reference to the medial-lateral and antero-posterior axes, respectively.

**Area:** Area is the spatial extent of a motor map in cm^2^. In general, area provides a measure of the spatial extent of excitability of muscle groups.

**Volume:** Volume is the MEP amplitude weighted spatial extent of a motor map in mV^∗^cm^2^.

Our literature review resulted in 132 unique articles. The mapping parameters used in these papers are outlined in Supplementary Table 1. As reliability was reported in a portion of studies only, this information is summarized separately in Supplementary Table 2 for studies reporting ICCs for outcome measures. This table is organized by muscle selection; however, other key mapping parameters are also denoted on the table to aid in identifying the application of specific methods. We have summarized trends in test–retest reliability based on ICC cut-offs (high > 0.75, moderate 0.5–0.74, low < 0.49; Portney, 1993) that appear when a common mapping parameter is employed in multiple studies.

We have focused our review to specific aspects of methodology not previously reported, including navigation, motor state, grid use, system factors, intensity and muscle selection and have organized results accordingly, examining benefits, limitations, tolerability and reliability within each section.

### Navigation

Navigation is how the location of the delivery of TMS pulses over the motor cortex is controlled and has evolved over time. It may be based on surface landmarks or magnetic resonance imaging (MRI), which may also be guided by a robotic system. Landmark-guided motor mapping involves the use of a flexible cap with regular grid markings, with stimulation occurring close to the grid markings. “Neuronavigated” TMS (nTMS) refers to the use of MRI co-registered with a targeting system (Singh et al., 1997), where the software used in conjunction with a targeting system displays the location of the stimulator coil relative to the brain in real time. The operator uses this signal to reduce position error prior to delivering a TMS pulse. Robot-controlled nTMS is a third navigation technique that utilizes a robotic arm to deliver TMS to specific pre-programmed grid sites.

Advantages of landmark-guided navigation include its ease of use, not requiring specialized equipment or imaging. The reliability of Centre of Gravity (CoG), map area and volume determined with landmark-guided navigation is moderate-to-high across studies which examine a variety of muscle groups (Supplementary Table 2; Mortifee et al., 1994; Oliveri et al., 1999; Malcolm et al., 2006; Plowman-Prine et al., 2008). nTMS, however, has been reported to be more accurate at stimulating a specific cortical target when compared to landmark-guided TMS (Leonard et al., 1998; Gugino et al., 2001; Julkunen et al., 2009), and can be used to facilitate stimulation of the same cortical sites weeks or months apart in longitudinal assessments. One group reported the magnitude of error associated with landmark guided scalp location error to be 1.4 cm on average vs. closer to 0.3 cm with guidance; coil twist error was also reported to be six-fold higher with landmark navigation (Gugino et al., 2001). This is likely due to the difficulty associated with an operator maintaining coil placement consistently in the absence of the online error-feedback provided by navigation systems. For example, average MEP amplitude at the hotspot (HS) is larger with nTMS compared to landmark-guided TMS, while its coefficient of variation is smaller (Julkunen et al., 2009). Variability in other MEP amplitude dependent map features such as CoG and volume are likely also improved with neuronavigation, as demonstrated by high between session reliability for a variety of measures (Supplementary Table 2). Robotic nTMS offers the same advantages of nTMS, but additionally reduces the positioning error introduced by a human operator. Robotic systems can deliver TMS with greater accuracy (in terms of smaller spatial errors in intended vs. actual coil placement) than hand-held nTMS or landmark-based guidance methods (Kantelhardt et al., 2010; Forster et al., 2012, 2014; Weiss et al., 2013; de Goede et al., 2018). In fact, one group reported positioning accuracy of 0.3 cm with a robotic system vs. 0.6 cm with manual positioning; this system also minimized repositioning errors (max error 1.0 cm between sessions vs. 2.4 cm manually; Ginhoux et al., 2013).

Limitations of landmark-guided techniques include the inability to provide information about map outcomes relative to actual cortical locations. Very little can be said about the true accuracy and reliability when this method is subject to errors in measurement and interpolation between different scalp landmarks, although one study reported increased (1.2 cm vs. 0.2 mm) cortical surface error (i.e., from the stimulated location to optimal cortical location) when landmark navigation was used (Gugino et al., 2001).

While a drawback of nTMS is the need for participants to complete a MRI scan in advance of testing (which increases the experimental cost and time), it is possible to complete nTMS using a standard Montreal Neurological Institute (MNI) “average brain,” with similar accuracy at stimulating scalp sites. Using the MNI atlas, however, no conclusion can be drawn about the underlying cortical features (Comeau, 2014) such as responses generated relative to the central sulcus, hand-knob, or other gyral structures. Other challenges include the symmetrical basis of the MNI atlas; whereas in reality, the handknob has marked differences in size and location between the two hemispheres, likely driven by hand dominance (Sun et al., 2012). The probabilistic location of the motor cortex, when mapped from the MNI atlas onto a subject’s MRI, was consistent with the M1 location derived via MRI-guided responses, however, with slightly higher position error (Sparing et al., 2008). No studies have compared performance of different commercially available robotic nTMS systems, however, there are discrepancies pertaining to ease of use. Some groups found robotic mapping to be faster (Grab et al., 2018) or similar (Ginhoux et al., 2013) to non-robotic mapping methods (Julkunen et al., 2009) with differences likely due to the use of a commercially available and a custom “proof-of-concept” robot, respectively.

**FIGURE 2:**
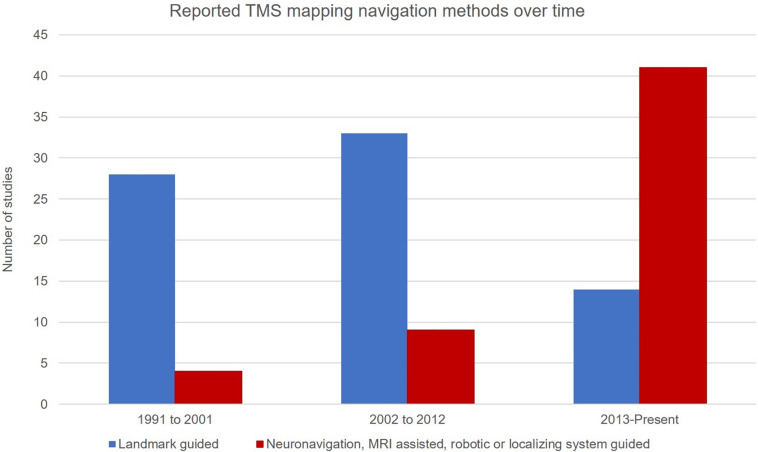
Studies reporting a particular navigation method by decade. Suggestive of a shift away from the use of landmark guided navigation (blue) in favor of neuronavigated methods and its subtypes (red).

Over the past 15 years there has been a shift from landmark-guided navigation to neuronavigation with a participant’s individual MRI (Figure 2). The enhanced stimulus localization capacity that is available with nTMS mapping has likely driven this change, ultimately resulting in greater experimental control. Localizing technology (Krings et al., 1997) combined with landmark-guided approaches, achieved typically through the use of an MNI atlas brain and standard neuronavigation equipment, provides the operator with specific coordinates of scalp landmarks in real time and can reduce the errors inherent in conventional landmark-guided techniques (Miranda et al., 1997). Recent use of electric field-guided mapping may assist localization, by displaying the cortical regions that are probabilistically expected to be targeted by the TMS coil (Gugino et al., 2001; Opitz et al., 2013; Thordstein et al., 2013; Schmidt et al., 2015).

Twelve studies report higher ICCs with the use of nTMS (with or without robotic control) compared to five which utilized landmark-guided navigation (Supplementary Table 2). While both techniques consistently produced moderate-to-high reliability for CoGx, more studies using landmark-guidance reported poor reliability in CoGy. Given the variability in the other mapping parameters used and outcome measures reported, it is difficult to draw broader conclusions about the comparative reliability of the two techniques alone. Reports of reduced response variation, high ICCs, and reduced position error in studies where MRI-guided neuronavigation is used support the application of the technique.

In summary, nTMS offers greater control of stimulus delivery compared to landmark-based methods. Furthermore, stimulation can be delivered relative to underlying cortical features such as the central sulcus, hand knob or other gyral features. While both landmark-guided and nTMS methods appear to be similar in reliability for several output measures, MEP variability is improved with nTMS. nTMS appears to be the superior method for cortical mapping, which can be improved further with robotic assistance. The obvious caveat is a situation where brain imaging is not available, in which case an MNI atlas can improve the accuracy of traditional landmark-guided TMS.

### Motor State

Motor state refers to the level of baseline activation in the muscles being mapped and includes both active and rest states. When mapping is at rest, the muscles of investigation are inactive and typically verified by surface EMG to ensure the absence of activation above a set threshold prior to delivery of the TMS pulse. In active motor mapping, a contraction of a certain size is maintained through EMG feedback, often relative to a maximum voluntary contraction. Because muscle activation increases cortical and spinal excitability, lower stimulation thresholds and larger MEPs result. Notably, 22/132 studies did not explicitly state whether mapping occurred at rest or during activation.

Higher stimulation intensities are generally required for activation in passive mapping; however, active mapping may negatively impact tolerability. Additional experimental controls are required for active mapping. For example, as MEP amplitude increases with activation, the degree of muscle activation must be matched to compare maps between individuals or within subjects following an intervention. EMG must be displayed to the participant to maintain a set contraction, requiring more equipment. Active mapping also requires independent activation and mapping of each muscle group under study, which can increase the total study duration compared to passive mapping. Furthermore, the participant must be capable of repeatedly producing a similar level of muscle activation, which may be challenging depending on what patterns of activity are required (Smith and Fisher, 2018). Passive mapping, however, allows the operator to examine many muscles simultaneously as they will all be under the same condition. It requires minimal engagement from participants, allowing it to be performed in children (Grab et al., 2018). This likely explains why it was used in 88/132 studies in this review.

Importantly, the maps produced under active and passive mapping are different: area and volume are larger during active mapping than passive mapping when similar stimulation intensities are used (Marconi et al., 2007; Ngomo et al., 2012). The single study that examined the reliability of active vs. passive state with otherwise similar methods found that while the reliability of map area is similar, map volume is more reliable during the active condition (Ngomo et al., 2012). Interestingly, imagining or viewing movement may also increase map area (Marconi et al., 2007), and the pattern of muscle activation (contraction vs. extension) may result in unique hotspots for the same muscle (Massé-Alarie et al., 2017). Overall, due to the ability to map several muscles simultaneously and the ease of maintaining muscles at rest, passive mapping is generally suggested, as reliability is similar to that produced from active maps. Notably for certain muscle groups in the trunk and lower limbs, activation thresholds are higher, and participant tolerability may necessitate that these muscle groups be mapped in the active state. This will be addressed in greater detail in the muscle selection section.

### Grid Use

Grids use a fixed pattern, or grid, of stimulation sites to deliver TMS and grid spacing is the distance between stimulation sites on the motor cortex. Traditionally, a coarse grid was used where the distance between stimulation sites is 1 cm or greater with finer grids being utilized where the spacing is less than 1 cm. 75 studies report using a square grid (i.e., two dimensions have equivalent spacing, and cover a set number of points), 51 report 1 cm^2^ grid spacing, 14 report grid spacing greater than 1 cm^2^, and 10 report grid spacing less than 1 cm^2^. This spacing is typically achieved using a flexible cap with stimulation sites marked on its surface for landmark-guided navigation, or a computer-generated grid through the use of nTMS. A grid may not be necessary to produce reliable maps if a participant’s MRI is used and nTMS is deployed for gridless mapping; in fact, six studies report forgoing the use of a grid entirely.

The major benefit of a coarse grid is that it requires less mapping time, compared to fine mapping, due to reduced density of test sites. Measures such as CoGx,y may become more accurate, in terms of distance to the true physiological CoG, with finer grid spacing (Brasil-Neto et al., 1992) as it allows for more responses to be averaged. Greater resolution can similarly influence HSx,y accuracy by providing additional test points from which to detect maxima. Of course this also provides the major drawback in that reduced resolution may decrease accuracy of the map generated, due to undersampling generating errors in map size or shape (Brasil-Neto et al., 1992). Area and volume on the other hand are more likely to be negatively influenced by variability at the map edges when more data points are included.

Gridless mapping does not require specialized setup, but software or manual examination may be used to iterate test sites based on prior measured responses (Meincke et al., 2016). Mapping without a grid is appealing in terms of its simplicity, which could reduce both experiment and setup time (Van De Ruit et al., 2015). However, gridless mapping risks missing cortical areas if electing to randomly stimulate within a pre-set area.

Of the 17 papers reporting ICCs, 12 reported the use of coarse grids, three reported fine grid use, and two did not report grid spacing size. Two groups found that the CoG and area reliability of a map were independent of grids. Randomly stimulating within a set area (Van De Ruit et al., 2015; Supplementary Table 2), or taking steps estimated to be 1 cm from a hotspot without a grid (Pitkanen et al., 2015), are both pseudo-random methods which produce reliable outcome measures (Supplementary Table 2; Van De Ruit et al., 2015), with ICC = 0.904 (Pitkanen et al., 2015) for agreement of area between maps produced with and without grid. While there were no studies directly comparing the reliability of fine and coarse grids, MEP amplitudes produced by stimuli 0.2 and 0.5 cm anterior, posterior, lateral and medial to a specific hand muscle hotspot were remarkably similar, suggesting that steps larger than 0.5 cm may be necessary to detect changes (de Goede et al., 2018). As mentioned above, coarse and fine grids may inversely influence the reliability of area or volume, and CoG or hotspot. Indeed, lower reliability in map area was reported in studies using fine grids compared to those using coarse grids; yet, higher reliability was found in CoGx,y, and HSx,y (Krause et al., 2006; Sparing et al., 2008; Weiss et al., 2013). Uncertainty surrounds the utility of a finer grid for achieving additional “resolution,” as enhanced reliability appears to be dependent upon the output measure examined. If interested in area and volume, coarse grids seem to be more reliable (Thickbroom et al., 1999) likely due to reported variability at map edges (Mortifee et al., 1994). Specific map features (i.e., HS, and CoG) appear to benefit from the averaging of additional sites via a coarse grid. Gridless mapping produces similar outcome measures to mapping with a grid (Julkunen et al., 2009), however, the reliability of this method has not been systematically examined.

There is a trade-off between total experimental time and resolution of produced maps, which is dependent on the use and coarseness of the stimulation grid. Mapping without a grid is faster (Van De Ruit et al., 2015), so theoretically better tolerated by participants. The additional experimental time required using a grid, and especially when testing with a finer grid with more test sites, may impact general excitability through boredom, fatigue or participant comfort. On the other hand, the use of a grid provides a level of standardization conducive to longitudinal or intervention-based testing and is more realistic for use in multi-centre studies. Gridless mapping remains a comparable alternative to mapping with a grid, however, this type of intervention seems to be best suited for individual test sessions rather than longitudinal assessments, and only when navigation with MRI is available.

Electric-field informed mapping, which accounts for probabilistic current spread within the brain in response to magnetic stimulation, could aid in selecting mapping intensities suitable for selected grid spacing (where areas of maximal induced current should not greatly exceed the grid space density). Computational modeling may similarly provide information regarding the number of discrete sites required to stimulate in order to produce reliable maps. Determining ICCs for coarse, fine and gridless mapping is also required to support the validity of each of these approaches for longitudinal studies.

### Stimulator System Factors

Transcranial magnetic stimulation motor mapping requires a coil to produce the magnetic field, and a stimulator to generate current in the coil. The vector relative to the motor cortex at which the stimulus is delivered, or “coil orientation” may influence the maps produced because it impacts which axons (interneurons or corticospinal projections neurons) are depolarized at a given stimulation intensity. Also, the waveform produced by the stimulator can be mono- or bi-phasic with these two patterns producing unique responses (Sommer et al., 2006).

**FIGURE 3:**
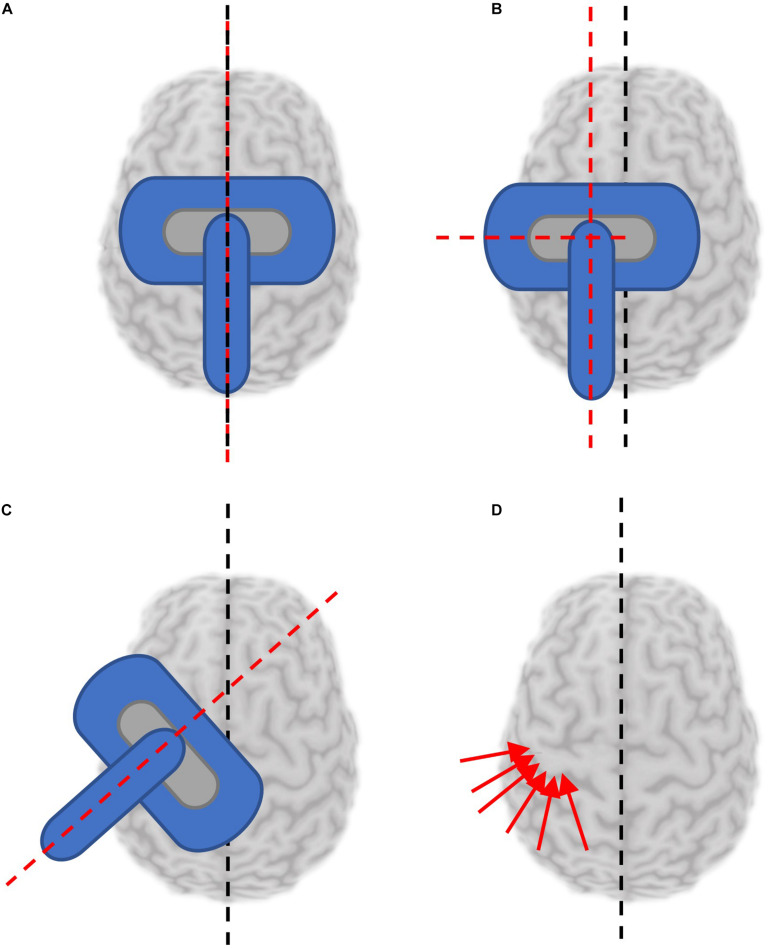
Reported coil orientations used for mapping. Black dashed line denotes orientation of presumed interhemispheric fissure. Red dashed lines or arrows denotes orientation of the TMS coil handle. **(A)** Coil coincident with and parallel to the sagittal plane. **(B)** Coil held parallel to the sagittal plane, at a distance from the interhemispheric fissure. **(C)** Coil held at an angle deviated from the parallel fissure (generally 45°). **(D)** Sulcus aligned mapping involves aligning the coil to be tangential to the sulcus at each stimulated location.

Mapping is most commonly completed with the coil orientation fixed relative to the head position (Supplementary Table 1). The coil is held to induce current in the brain consistently in the sagittal plane, along the anterior-posterior axis (Figures 3A,B) for mapping lower extremity muscles, or at typically 45° from the sagittal plane (Figure 3C) when mapping upper extremity muscles. Sulcus-aligned mapping refers to mapping with the coil maintained perpendicular to the cortical sulci (Figure 3D) rather than to the axis of the skull. One modification to this method involves coarse mapping around a particular sulcus at a fixed orientation to find a muscle hotspot, and then modifying coil orientation to find the orientation producing the largest MEP. In fact, the CoGx coordinate shifts with coil orientation (Van De Ruit et al., 2015).

Sagittal coil positioning is relatively simple to maintain with or without neuronavigation tools, however, maintaining a coil position fixed 45° to the midline is more challenging and results in MEP amplitudes with greater variability. In fact, the coefficient of variation was reported to be almost 1.3 times higher mapping the same muscle representation without navigation compared to navigated sulcus aligned mapping (Julkunen et al., 2009). When performing sulcus-aligned mapping, the coil trajectory varies throughout testing to maintain a perpendicular orientation (Weiss et al., 2013). This technique requires neuronavigation with a participant’s individual MRI and an experienced operator. While it might produce maps which are more accurate to an individual, it is more difficult to perform, and may complicate inter-participant comparisons. Sulcus-aligned mapping, unlike fixed orientation mapping, takes unique physiology into consideration. Interestingly, sulcus-aligned mapping of the first dorsal interosseous (FDI) muscle produced a CoG on average 0.4 cm away from the CoG produced with variable coil orientations suggesting that sulcus-aligned mapping may reduce the error by this much (Zdunczyk et al., 2013).

Various coil orientations produce different results for some outcome measures; for example, one study found that RMT but not CoG was influenced by coil orientation (Niyazov et al., 2005). Additionally, there is considerable inter-individual variability in “optimal” coil orientation at any given grid site: one site may maximally activate the muscle under study when aligned nearly perpendicular to the central sulcus, while another grid site in the same individual maximally activates the same muscle when parallel to the central sulcus (Reijonen et al., 2020).

Six studies reported reliability for coil orientation parallel to the sagittal plane, all with moderate or high reliability for measures of area and volume. ICCs for CoGx and CoGy among this group was also moderate to high, with only one group reporting low reliability in CoGy for FDI. Three studies reported reliability for coil orientation perpendicular to the central sulcus, mapping parallel to the sagittal plane (Supplementary Table 2). All had high or moderate-high reliability for CoGy and HSy. Sulcus-aligned mapping allows the motor representations of muscles which are located at adjacent cortical sites to be distinguished (e.g., the hotspot location of abductor digiti minimi vs. FDI) and are indistinguishable when using a 45° to midline paradigm (Raffin et al., 2015). One study reported reliability of sulcus-aligned mapping longitudinally using ICCs, with very high correlation between sessions in the targeted muscle (extensor digitorum communis; Schaworonkow et al., 2019).

Overall, commonly used coil orientations are each best suited to particular applications. Where nTMS is not available to help with sulcal alignment, fixed-orientation mapping (ideally parallel to the midline) should be utilized to minimize variability related to coil position while still producing outcome measures with moderate to high reliability. Standardized coil orientation also allows for between-subject comparisons more readily than variable coil orientation, which is individualized. Sulcus aligned mapping has the advantage of being able to distinguish between muscle groups which are represented at adjacent cortical sites to a greater extent than fixed orientation mapping. If individual participant imaging is available, and the study aims are to observe phenomenon within an individual (ideally at one time point), sulcus-aligned mapping is advantageous as it provides responses relative to underlying gyral anatomy.

Finally, the configuration of the TMS coil, or coil design, controls the characteristics of the magnetic field it produces. Stimulator output may not influence whether or not a site is responsive (Stephani et al., 2016), but potentially the characteristics (amplitude, latency, waveform) of the MEP elicited. Common coil designs used for mapping include figure-of-8, circular and double-cone; each produces a magnetic field with a different degree of focality and depth (Thielscher and Kammer, 2004; Deng et al., 2013). The impact of using different coils on mapping of the same muscles has not been systematically studied, however, both output and coil design are likely to impact map outcome measures and thus should be kept consistent within an experiment.

### Intensity

The intensity of TMS utilized will significantly impact motor maps generated. At a given location, MEP amplitude will increase when increasing TMS intensities above motor threshold until a plateau is reached, known as the stimulus-response curve. Increasing TMS intensity will reduce focality of the map as more of the muscle map is activated. In fact, modeling studies suggest that even “focal” figure-of-8 coils activate 10–35 cm^2^ of cortex at 130 and 200% of a calculated threshold, respectively, (Thielscher and Kammer, 2004). Intensity is commonly selected as a function of the RMT, which can be derived using a variety of methods (Kallioniemi and Julkunen, 2016) and is not the subject of the current review. Stimulation intensities of 110% (49/132 papers) or 120% (51/132) of the motor threshold were most commonly selected to produce motor maps in the literature. These percentages relate to active motor threshold for mapping activated muscles or RMT for mapping muscles at rest.

Selecting an inappropriately low intensity may cause certain features of the map to be missed as transient fluctuations in excitability could reduce the probability and amplitude of an MEP, thereby influencing hotspot/CoG location, area, and volume (Brown et al., 2017). On the other hand, selecting an inappropriately high intensity may result in current spread producing larger map areas and volumes. CoG and hotspot may become difficult to distinguish, whereby MEPs from multiple locations are at the maximal level for that muscle as current spreads to make inactive sites appear to produce MEPs (Thickbroom et al., 1998). No systematic evaluation of output measures produced using common test intensities has been done. No longitudinal study investigating the reliability of such features over time has been published. Yet, some efforts have been made to understand the effects of intensities on motor maps. One group found good correlation between maps produced at 105% of threshold using a 50 and 500 μV threshold for MEP amplitude, but not for a 300 μV amplitude (Lucente et al., 2018). Overall this suggests that selection of a mapping intensity, when informed by stimulus-response curve characteristics, may reduce response variability compared to simply selecting intensity in relation to RMT.

Kallioniemi and Julkunen (2016) examined aspects of response variability by using standard test intensities (110 and 120%RMT), and comparing responses produced at those intensities to responses obtained using the upper-threshold (UT) intensity value (Mills and Nithi, 1997). The UT is the lowest intensity at which 100% of stimulations result in an MEP of at least 50 μV; it can be conceptualized as a point on the “plateau” of a sigmoidal stimulus response curve [see Figure 5 in Kallioniemi and Julkunen (2016)]. Mapping at this intensity therefore produces more similar conditions for comparisons between participants rather than applying standard multiples of the RMT (110, 120%, etc.), which could place the test intensity in each subject at different positions along the stimulus response curve. Overall, they found that responses at 110%RMT better approximated responses at UT than 120%RMT [i.e., the mean stimulator output was not significantly different between 110%RMT (43% Maximum Stimulator Output (MSO)] and UT (44%MSO), while 120% was significantly higher (47%MSO), which suggests that 110%RMT may be a better intensity selection than 120% given the sigmoidal stimulus-response relationship.

In the absence of studies critically examining reliability and features of mapping outcome measures produced using a variety of stimulus intensities, mapping at 110% seems to strike a sufficient balance between participant tolerability, minimizing likelihood of excessive current spread, and consideration of stimulus response properties.

### Mapped Muscles

While the decision of which muscle(s) to map is often driven by the research question, there are other considerations which may drive selection of muscle. An overview of the muscles mapped in all of the studies we reviewed and their abbreviations is included in Supplementary Table 1. Muscle selection may also be influenced by tolerability, which varies between muscles. The ability to map several muscles simultaneously may also be desirable.

In general, muscles of the hand and upper limbs can be mapped with high tolerability due to the location of the representation of these muscles at more superficial cortical sites. 111 of 132 the studies we reviewed included muscles of the hand or upper limb. A number of upper limb muscles have been included in reliability assessments, with abductor pollicis brevis (APB) and FDI being investigated in the greatest number of trials reporting ICCs (Supplementary Table 2). Lower limb (Weiss et al., 2013) and trunk (Ferbert et al., 1992) muscles have higher thresholds than upper limb and face muscles, requiring higher stimulation intensities or specialized coils due to the depth of the motor homunculus and the strength of corticospinal connections. These higher intensities can be more uncomfortable due to activation of sites beyond the target muscle. Stimulation sites more lateral than the hand knob, often required for mapping face and throat musculature, can uncomfortably activate facial nerves and muscles directly. Certain muscle groups also require specialized electrodes to record muscle activity, such as swallowed electrodes (Aziz et al., 1996; Hamdy et al., 1996; Plowman-Prine et al., 2008) endoscopically assisted placement (Rödel et al., 2004), or needle electrodes (Rödel et al., 2004) for throat and cranial muscle groups (Meincke et al., 2018). Not only do these electrodes influence participant comfort but the muscles are bilaterally represented (Aziz et al., 1996) so additional testing time would be required to map both hemispheres.

In general, tolerability is an important factor when selecting muscles to be mapped. In instances where a global phenomenon is of interest, hand and upper limb muscles are the best mapping targets. More accounts of reliability of these muscle groups, the FDI and APB in particular (refer to Supplementary Table 2) already exist and may assist in verifying baseline measures. When muscles of the trunk or lower limb must be mapped, ideally this is done in the active state to improve tolerability. However, the level of activation must be verified throughout trials by monitoring EMG to insure that activation is achieved, as well as monitoring participant fatigue which may contribute to inability to complete active trials (Smith and Fisher, 2018). When muscles of the face and throat must be mapped, activation may also assist with decreasing threshold and improving tolerability.

## Discussion

A detailed understanding of mapping parameters and their influence on outcome measures can improve the likelihood of evaluating relevant physiology and detecting change in any given study. We identified a substantial lack of consistency among methodology used for mapping, which has persisted in the literature since the earliest experiments. Given the level of detail available in most methods sections, it would be challenging for any researcher to replicate an experiment as many elements would need to be assumed. If we restricted the studies included in the review to those meeting the quality standards proposed by Chipchase et al. (2012) we would have been left with very few studies to discuss. To this end, a systematic review, which considers study quality, would have been limiting in its ability to draw conclusions about this important topic required to advance the field.

We omitted two mapping parameters in this review: threshold determination and the number of stimuli required for assessment. This was necessary to constrain the scope of this review with the understanding that important efforts have already been made to address these parameters. Cavaleri et al. (2017) provides a robust overview of the number of stimuli required to assess function while limiting variability, ultimately recommending a minimum of five stimuli at a single site for optimal within-session reliability of MEP amplitude, and 10 or more for optimal inter-session reliability. The International Federation of Clinical Neurophysiology suggested that adaptive methods of thresholding (selection based on probabilistic response) provide a faster and more accurate method of thresholding (Rossini et al., 2015) compared to other techniques, and subsequent work has characterized (Dissanayaka et al., 2018) and applied (Julkunen, 2019) such adaptive techniques. Other methodological factors broadly applicable to TMS of the motor system which should be reported and controlled for in any TMS study, as identified by Chipchase et al. (2012) include the positioning of EMG electrodes, quantification of relaxation (or activity) in tested muscles and prior activity, the time between MEP trials, total number of MEP trials, and time between test days for intersession assessments. To our knowledge these factors have not been systematically reviewed elsewhere, so beyond recommending that these factors be kept consistent across participants, little more can be suggested. Notably, at least one group has suggested that reliable maps can be produced with 1 s intervals between MEP trials (Van De Ruit et al., 2015), however, the assumption that MEP amplitudes are time invariant has been demonstrated to be inaccurate (Julkunen et al., 2012), lending further support to the suggestion that a minimum number of MEPs per site be collected. Recently, a mathematical framework to aid investigators in establishing the optimal total number of MEPs (to strike a balance between variability and experiment duration) has been proposed (Ammann et al., 2020), and is poised to aid in this aspect of experimental design.

The results of this review suggest the use of a navigation system, the use and size of a grid are relevant parameters to report in the methods section of any study reporting mapping outcomes, in addition to important criteria outlined previously (Chipchase et al., 2012). Many of the sample sizes of the studies were low despite the introduction of technological innovations which have improved ease of use. As a result reliability found in one study may not be reproducible in a future study as replicating incompletely described methods is not possible.

Based on our review, we have created a summary table (Table 1) which might aid experimenters in selecting mapping parameters, based on the outcome measure(s) of interest and feasibility. First, for interventional and longitudinal studies, multiple baseline outcome measures should be collected whenever possible to establish the reliability of the measures with the specific testing parameters, as well as the appropriate sample size. Additionally, methods should be highly detailed so that results can be reproduced outside of the reporting laboratory.

**TABLE 1:** Effect of mapping parameters on outcome measures and feasibility.

	Outcome measure considerations	Feasibility considerations
Navigation	• nTMS facilitates consistent delivery of TMS to the same cortical sites • Important for MEP amplitude dependent outcome measures (hotspot, CoG, and volume) to minimize variability	• Landmark guided TMS is expedient; however, no conclusions about underlying anatomy may be drawn • Robotic nTMS is easier for the operator in studies requiring long mapping sessions
Motor state	• Active mapping produces larger map areas and volumes regardless of intensity • CoG relies on spatial averaging of responses and therefore is more robust, in theory, to motor state	• Active mapping may improve tolerability of TMS as it generally reduces the intensity required • Passive mapping is easier to implement than active mapping, which requires constant subject attention and participation to maintain contractions
Grid use	• Gridless mapping is suitable for CoG, hotspot determination by using spatial averaging across multiple responses • For all output measures, use of a grid is advantageous for longitudinal and between groups assessments	• Gridless mapping outcome measures should be interpreted carefully in case cortical sites are missed • When time allows, fine grid spacing should be used, as spacing can be down-sampled during analysis
Stimulator system factors	• Sulcus aligned mapping generally less informative for area and volume measures • Sulcus aligned mapping is useful for within subject hotspot and CoG assessments	• When nTMS is not available, fixed orientation mapping decreases variability while preserving reliability • Fixed orientation mapping is most suitable for longitudinal or between subject comparisons
Intensity	• Area and volume are influenced by current spread and therefore are intensity dependent • CoG and hotspot are generally robust to the effects of intensity	• Stimulation intensity influences participant tolerability • 110% RMT is most commonly used for passive mapping and may be best compromise between reliability and ease of use
Muscle selection	• More studies have reported ICCs for studies of the upper limb • Depends on research question	• Hand and upper limb muscles are easiest to map due to superficial representation • Bilateral representation of some muscles must be considered

“Neuronavigated” TMS or robot-assisted nTMS mapping may enhance ease of use and accuracy in stimulus localization. When nTMS is available, sulcus aligned mapping aids in determining the differences in mapping outcome measures relevant within an individual, while fixed orientation mapping should be deployed to examine inter-individual changes. Where trial length, study population and muscle of interest allows, active mapping can reduce variability in MEP response. It is unclear whether 0.5 or 1 cm grid spacing is more appropriate in general mapping applications. Where time is not a factor, mapping with 0.5 cm spacing is advantageous as the experimenters can opt to “down-sample” to 1 cm. Test equipment (i.e., stimulator and coil design) is a factor to consider when interpreting outcome measures; we recommend that authors maintain consistent equipment during a study and consider the influence that their test apparatus could reasonably have made on their reported measures (Thielscher and Kammer, 2004; Deng et al., 2013). Few studies have compared maps produced at various stimulation intensities systematically, and we strongly suggest that such a study be performed. In the absence of such a study, mapping at a high intensity when trying to reduce variability is an acceptable approach if MEP saturation is not a concern, such as when CoG or HS make up the key outcome measures, rather than volume or area. Alternatively, the lowest possible intensity to reliably produce a MEP (i.e., 110% – above the 50 μV threshold) may be selected, if looking to examine features while avoiding saturation of the MEP response with current spread. Muscle selection naturally follows the research question, however, when examining motor cortex excitability generally, hand muscles may be tested preferentially for ease and tolerability.

It is also worth noting that new rapidly developing techniques may aid in the reproducibility of motor mapping with TMS. For example, electric field mapping has the potential to provide additional probabilistic inferences related to TMS outcome measures by supplying the operator with presumed tissues activated by TMS electric field at each grid site. It has the potential to challenge the physiological meaning of many accepted TMS outcome measures by defining cortical areas most likely to be activated by TMS based on the distribution of the electric field in underlying cortical geometry, and may improve TMS localization (Pitkänen et al., 2017; Laakso et al., 2018; Weise et al., 2020). Electric field mapping is poised to provide robust explanation for at least a portion of the variability we explored in this review. However, the users of electrical field mapping must still select and report a variety of parameters. Electroencephalography combined with TMS is another technology being more widely incorporated into TMS studies which is similarly likely to improve variability in mapping outcome measures (Schaworonkow et al., 2019).

Transcranial magnetic stimulation mapping is a valuable tool with emerging clinical and research applications. Careful selection of the mapping parameters and relevant outcome measures is an important step in refining the technique and producing meaningful and reproducible outcomes. In many cases, further research with a specific set of mapping parameters is needed to confirm validity and reliability for a given application. This work will ensure multi-centre or longitudinal studies are conducted using equivalent techniques, reducing operator bias and improving the quality of data produced. Initiatives such as the Big TMS Data Collaboration appear to be well positioned to tackle some of these challenges, as has already been demonstrated for other TMS applications (Corp et al., 2020). This will become increasingly important as quantitative analysis tools, like TMSmap (Novikov et al., 2018) and Neuromeasure (Gerber et al., 2019) will improve ease of analysis, but may facilitate less consideration of the physiological meaning of outcome measures. As with any tool, consideration of participant comfort and other practicalities are also important factors. Acknowledging MEP variability and attributing it to specific controllable elements can help drive innovation in commercial device development, as well as developing new tools for analysis. We hope this review provides the reader with a greater ability to design and interpret TMS mapping experiments.

## Author Contributions

RS: project conception, organization and execution, design and execution of analysis, and writing of the first draft. DM: review and critique of analysis, and manuscript review and critique. ZK: project conception, review and critique of analysis and manuscript review and critique. EC: project conception and organization, design and critique of analysis, and manuscript review and critique. All authors contributed to the article and approved the submitted version.

## Conflict of Interest

The authors declare that the research was conducted in the absence of any commercial or financial relationships that could be construed as a potential conflict of interest.

## Publisher’s Note

All claims expressed in this article are solely those of the authors and do not necessarily represent those of their affiliated organizations, or those of the publisher, the editors and the reviewers. Any product that may be evaluated in this article, or claim that may be made by its manufacturer, is not guaranteed or endorsed by the publisher.
