# Supplementary material for: TMS Motor Mapping Methodology and Reliability: A Structured Review
Source: Front Neurosci. 2021 Aug 19;15:709368. doi: 10.3389/fnins.2021.709368 (PMC8417420; doi:10.3389/fnins.2021.709368)
Supplement: Supplementary file 1 [file Table_1.docx]

|  | **Supplementary Table 1: TMS Motor Mapping Parameters in Reviewed Literature** | | | | | | | |
| --- | --- | --- | --- | --- | --- | --- | --- | --- |
|  | **Study** | **Population*** | **Navigation Method** | **Motor State** | **Use of grid** | **Muscles** | **Coil Orientation** | **Intensity** |
|  | (Ahdab, Ayache et al. 2014) | N= 50 healthy controls | MRI guided neuronavigation | Not stated | 12 squares via MRI coordinates of reliable landmarks | FDI | Not stated | 120% FDI RMT |
|  | (Ahdab, Ayache et al. 2016) | N = 12 healthy controls | MRI guided neuronavigation | At rest verified by EMG | 0.5cm spacing | FDI, in subset ADM, APB | Perpendicular to the central sulcus | 120% RMT of FDI |
|  | (Aziz, Rothwell et al. 1996) | N = 10 healthy controls | Landmark guided | Not stated, assumed at rest | 10 x 10 grid with 1cm squares | APB, esophagus | Parallel to sagittal plane | 0.4T > RMT of each muscle |
|  | (Bashir, Perez et al. 2013) | N= 10 young healthy controls | MRI guided neuronavigation | Not stated | 1 grid point x 8 orientations | FDI, APB, ADM | 8 cardinal directions examined | 110% FDI, APB, ADM RMT |
|  | (Bastings, Gage et al. 1998) | N =4 healthy controls | Landmark navigated | Not stated, assumed at rest | Grid with 1cm spacing | FDI | Not stated | 110% RMT |
|  | (Bastings, Greenberg et al. 2002) | N = 12 healthy controls | Landmark guided | At rest | Grid with 1 cm spacing | FDI | Parallel to sagittal plane | 110% RMT |
|  | (Bernard and Seidler 2012) | N = 16 healthy controls and 17 older controls | Landmark guided | At rest verified by EMG | 6 x 6 grid with 1.2cm spacing | FDI | Not stated | 110% RMT |
|  | (Bernard and Seidler 2012) | N = 33 young, N = 23 age > 70 | Landmark guided | At rest verified by EMG | 6 x6 grid with 1.2cm spacing | FDI | 45 degrees from sagittal plane | 110% RMT |
|  | (Boroojerdi, Foltys et al. 1999) | N = 4 healthy controls | Landmark guided | At rest | 5 x 5 grid with 1.5cm spacing | FDI, APB, ADM | 45 degrees from sagittal plane | 0.5-1.0 mV response in APB |
|  | (Brasil-Neto, McShane et al. 1992) | N= 5 healthy controls | Landmark navigated | At rest verified by EMG | 0.5 or 1cm apart along coronal line lateral to Cz | DEL, BB, FCR, APB | Parallel to sagittal plane | 100% MSO |
|  | (Brogårdh, Johansson et al. 2010) | N = 30 healthy controls | Landmark guided | At rest | A grid with 1cm spacing | APB | Parallel to sagittal plane | 120% RMT |
|  | (Brouwer and Hopkins-Rosseel 1997) | N = 12 healthy controls | Landmark guided | At rest and with 5%-10% of max voluntary contraction | A grid with 1.5cm spacing | BB, DEL, TRI | Not stated | 100% MSO |
|  | (Buick, Kennedy et al. 2016) | N = 7 young healthy controls | Landmark guided | At rest verified by EMG | 5 x 5 grid with 1cm spacing | APB, FDI, ADM | 45 degrees from sagittal plane | 120% RMT of ADM |
|  | (Cavaleri, Chipchase et al. 2020) | N = 30 healthy controls | Neuronavigation without MRI | Active at ~20% MVC | 5 x 7 grid with 1cm spacing | Lumbar erector spinae | parallel to saggital plane | 120% AMT |
|  | (Cavaleri, Schabrun et al. 2018) | N = 20 young healthy controls | Neuronavigation without MRI | At rest verified by EMG | 6 x 6 grid with 1cm spacing, and random walk method at a variety of ISIs | FDI, APB, ECR | 45 degrees from sagittal plane | 120% ECR RMT |
|  | (Chernyavskiy, Sinitsyn et al. 2019) | N = 15 healthy controls | MRI guided neuronavigation | Not stated, assumed at rest | “determined individually considering previous responses” | EDC, FDS | Perpendicular to the central sulcus | 110% of motor threshold |
|  | (Chieffo, Straffi et al. 2016) | N = 12 young healthy controls | Landmark navigated | At rest verified by EMG | A grid with 1cm spacing. | APB, ADM, ECR | Handle oriented ‘posteriorly’ | 115% APB and/or ADM RMT |
|  | (Cicinelli, Traversa et al. 1997) | N = 20 healthy controls | Landmark guided | At rest | 11 sites per hemisphere, average of 2 cm between points | ADM | Approximately perpendicular to central sulcus | RMT + 10% MSO |
|  | (Cicinelli, Traversa et al. 1997) | N= 20 healthy controls | Landmark navigated | At rest | 11 sites per hemisphere, average of 2 cm between points | ADM | Approximately perpendicular to central sulcus | ~110% ADM RMT |
|  | (Classen, Knorr et al. 1998) | N= 11 young healthy controls | Landmark navigated | At rest and active verified by EMG | 7 x 7 x 1cm grid | FDI, AH, MM | Parallel to sagittal plane | 120% FDI, AH, MM RMT |
|  | (Corneal, Butler et al. 2005) | N= 11 healthy controls | Landmark navigated | At rest verified by EMG | Grid with 1cm spacing | APB | Parallel to the sagittal plane | 110% APB RMT |
|  | (Davies 2020) | N = 16 young healthy controls | Neuronavigated without MRI guidance | At rest, verified by EMG | 9 x 7 grid with 1cm spacing | rectus femoris, vastus lateralis, vastus medialis, medial hamstring, lateral hamstring, medial gastrocnemius, lateral gastrocnemius | Parallel to sagittal plane | "Consistent MEPs in all muscles while remaining tolerable" |
|  | (de Goede, ter Braack et al. 2018) | N = 8 young healthy controls | Robot assisted neuronavigation | At rest | 0.2cm and 0.5cm away from ADM hotspot along Anterior-Posterior and Medial-Lateral directions | ADM | 45 degrees from sagittal plane | 110% ADM RMT |
|  | (Delvaux, Alagona et al. 2003) | N = 20 healthy controls | Landmark guided | At rest verified by EMG | Grid with 1cm spacing | FDI | Parallel to sagittal place | 125% of RMT |
|  | (Devanne, Cassim et al. 2006) | N = 17 healthy controls | Landmark guided | Active mapping (10% MVC) | Grid with 1cm spacing | FDI, ECR, AD | Parallel to sagittal place | 120% AMT or 110% AMT |
|  | (Engelhardt and Picht 2020) | N = 20 | MRI guided neuronavigation | At rest verified by EMG | Not mentioned | APB | Not stated | 105% RMT |
|  | (Elgueta-Cancino, Schabrun et al. 2018) | N = 20 young, healthy controls | Neuronavigated without MRI guidance | During active contraction monitored by EMG | 5 x 7 cm grid | Erector spinae | Parallel to the sagittal plane | 100% MSO |
|  | (Ferbert, Caramia et al. 1992) | N = 9 healthy controls | Landmark guided | During active contraction monitored by EMG | 2 cm spacing | Erector spinae | Parallel to the sagittal plane | 100% MSO and below |
|  | (Forner-Cordero, Steyvers et al. 2008) | N = 11 healthy controls | Landmark guided | At rest | 1cm x 1cm grid | FCR, ECR | 45 degrees from sagittal plane | 120% FCR RMT |
|  | (Forster, Senft et al. 2012) | N= 5 healthy controls | MRI guided neuronavigation | Not stated | 19 x 19 square grid (spacing not mentioned) | FDI, APB, EDC, TA, AH | Perpendicular to the central sulcus | 110% FDI RMT |
|  | (Forster, Limbart et al. 2014) | N=12 healthy controls | MRI guided neuronavigation | At rest verified by EMG | Not stated | FDI, TA, AH, APB, EDC | Perpendicular to the mapped gyrus | 110% FDI RMT for upper limb, and 110% TA RMT for lower lumb |
|  | (Freund, Rothwell et al. 2011) | N=14 healthy controls | Neuronavigated without MRI guidance | Active mapping (10% MVC) | 8 x 9 grid with 1cm spacing | EDC | 45 degrees from sagittal plane | 110% AMT |
|  | (Gallas, Marie et al. 2009) | N = 9 young healthy controls | Landmark navigated | At rest | 10 x 10 grid with 2cm spacing centered at vertex | Mylohyoid, APB | Parallel to the sagittal plane | 120% RMT |
|  | (Ginhoux, Renaud et al. 2013) | N= 10 healthy controls | MRI guided neuronavigation, and MRI guided neuronavigation with robot | At rest and active | 10 x 10 grid with 1cm spacing | APB | Not stated | Not stated |
|  | (Green, Cheong et al. 2015) | N = 15 healthy controls | Landmark guided | At rest, verified by EMG | 5 x 5 square grid centered around the FDI hotspot with 1cm spacing | FDI | 45 degrees from sagittal plane | 110% RMT |
|  | (Guerra, Petrichella et al. 2015) | N=9 healthy controls | Landmark guided | At rest, in supine position | 7 x 7 grid with 1cm spacing, centered at the hotspot | ECD, ADM | Not stated | 110% RMT |
|  | (Hamdy, Aziz et al. 1996) | N = 20 healthy controls | Landmark guided | Not stated | 12cm x 9 cm grid with 2cm anteroposterior spacing, 1cm mediolateral spacing | Mylohyoid, pharyngeal, esophageal | Not stated | 0.4T above RMT for simultaneous activation of all 3 muscles |
|  | (Herwig, Kölbel et al. 2002) | N = 8 healthy controls | MRI-guided neuronavigation | At rest | 0.5 x 0.5 cm grid | APB | Perpendicular to sagittal plane | 120% RMT or 110% RMT |
|  | (Inuggi, Filippi et al. 2010) | N = 7 healthy controls | Landmark guided | Not stated, assumed at rest | 4 x 5 grid with 1cm spacing | APB | Not stated | 115% RMT |
|  | (Jonker, van der Vliet et al. 2019) | N = 21 healthy controls | Neuronavigated without MRI guidance | At rest verified by EMG | 1cm x 1cm for grid, or 8 lines straight out from hotspot, clockwise ellipsoid around these lines, then random pulses within the ellipsoid | FDI | 45 degrees from sagittal plane | 110% FDI RMT |
|  | (Jono, Chujo et al. 2015) | N = 11 healthy controls | Landmark guided | Active, at ~10% of maximum verified by EMG | 5 x 5 grid with 1cm spacing | FDI, ADM | 45 degrees from sagittal plane | 100% RMT |
|  | (Julkunen, Saisanen et al. 2009) | N= 8 healthy controls | MRI guided neuronavigation and landmark guided | Not stated | No grid used | APB | 45 degrees to the nasion-inion (landmark guided) or perpendicular to the central sulcus (neuronavigated) | Not stated, ‘somewhat higher than MT’ |
|  | (Julkunen 2014) | N = 6 young healthy controls | MRI guided neuronavigation | At rest, verified by EMG | With and without grid | APB, ADM | Not stated, assumed at the optimal coil orientation | 110% RMT |
|  | (Kallioniemi, Pitkanen et al. 2015) | N= 10 young healthy controls | Neuronavigation without MRI guidance | At rest verified by EMG | With and without a grid (0.5cm spacing) | FDI, APB, ADM | Perpendicular to the nearest sulcus | 110% FDI RMT |
|  | (Kallioniemi, Pitkänen et al. 2016) | N = 10 healthy controls | MRI guided neuronavigation | At rest | Grid with cell size 0.5 x 0.5 cm^2^ | FDI, APB, ADM | Perpendicular to the nearest sulcus | 110% RMT |
|  | (Kallioniemi and Julkunen 2016) | N= 12 healthy controls | MRI guided neuronavigation | At rest | Grid used, spacing not mentioned, extended until no response | FDI, ADM, APB | Stated for thresholding but not mapping procedure | 110% RMT, 120% RMT and UT |
|  | (Kantelhardt, Fadini et al. 2010) | N= 1 healthy controls | MRI guided neuronavigation with robotic assistance | Not stated | 0.8cm steps outward from the hand knob in all directions | brachioradialis, APB, ADM | 45 degrees to the sagittal plane | 40-45% MSO |
|  | (Komoda, Iida et al. 2015) | N = 16 young healthy controls | Landmark guided | FDI, tongue at rest, MM during active contraction | Grid with 1cm spacing | FDI, left tongue, right tongue MM, FDI | 45 degrees from sagittal plane | 120% RMT for each muscle |
|  | (Kosik, Terada et al. 2017) | N = 16 healthy controls | Landmark guided | During active contraction | 6cm x 6 cm grid | Fibularis longus | Not stated, assumed parallel to sagittal plane | 100% AMT |
|  | (Kothari, Svensson et al. 2013) | N = 48 | Landmark guided | At rest | 1 x 1 cm2 grid | Right tongue dorsum and FDI | 45 degrees from sagittal plane | 120% RMT |
|  | (Kraus and Gharabaghi 2015) | N= 10 healthy young controls | MRI guided neuronavigation | Not stated | ~0.3cm spacing, extended until no response measured | EDC | Orientation producing largest response at the hotspot | 110% EDC RMT |
|  | (Kraus and Gharabaghi 2016) | N= 12 healthy young controls | MRI guided neuronavigation | Not stated | ~0.3cm spacing extended until no response measured | EDC | Orientation producing largest response at the hotspot | 110% EDC RMT |
|  | (Krause, Förderreuther et al. 2006) | N = 10 healthy controls | Landmark guided | At rest | 1cm spacing | EDC | 45 degrees from sagittal plane | 120% RMT |
|  | (Krings, Naujokat et al. 1998) | N = 18 healthy controls | MRI guided neuronavigation | At rest, verified by EMG | 10 sites in a line along the central sulcus spaced 1cm apart | AH, TA, DEL, TRI, BB, FCR, ADM, FDI, APB, QF, gastrocnemius, serratus anterior | Perpendicular to the central sulcus | 110% and 120% of RMT |
|  | (Krings, Buchbinder et al. 1997) | N = 3 healthy controls | MRI guided neuronavigation | At rest, verified by EMG | 4 x 6 scalp sites with ~1cm spacing | FCR, FDI | Perpendicular to the central sulcus | 110% RMT |
|  | (Labyt, Houdayer et al. 2007) | N = 8 healthy controls | Landmark guided | At rest | 1.5cm spacing | APB | 45 degrees to sagittal plane | 120% RMT |
|  | (Lewis and Byblow 2004) | N = 12 healthy controls | Landmark guided | At rest | 1cm spacing | FCR | 45 degrees to sagittal plane | 110% RMT |
|  | (Lewis, Signal et al. 2014) | N = 13 healthy controls | Landmark guided | Active verified by EMG | Grid with 1cm spacing | Soleus | Parallel to sagittal plane | 120% RMT |
|  | (Li, Li et al. 2020) | N= 51 healthy controls | Landmark guided | At rest verified by EMG | Grid with 1cm spacing | Pharynx | Parallel to the sagittal plane | 120% RMT |
|  | (Littmann, McHenry et al. 2013) | N= 6 healthy controls | Navigation using digitized landmarks | At rest verified by EMG | 15-point grid based on compass ordinates with 1cm spacing | FDI | 45 degrees to sagittal plane | 120% FDI RMT |
|  | (Lu, Baad-Hansen et al. 2013) | N = 15 young healthy controls | Landmark guided | During active contraction for MM, at rest for FDI | Grid with 1cm spacing | FDI, MM | 45 degrees from sagittal plane | 120% of AMT or MT |
|  | (Lucente, Lam et al. 2017) | N= 7 healthy controls | MRI guided neuronavigation | At rest | Spacing < 1cm | FDI | Perpendicular to stimulated gyri | 105% of 50µV, 300µV and 500µV RMT |
|  | (Malcolm, Triggs et al. 2006) | N= 20 healthy controls | Landmark navigated | At rest | 5 x 5 grid with 1cm spacing, extended until no response measured | FDI, APB, EDC, FCR | Parallel to sagittal plane | 115% FDI, APB, EDC, FCR RMT |
|  | (Marconi, Pecchioli et al. 2007) | N = 8 healthy controls | Landmark guided | At rest and during active contraction | 10.5 x 10.5cm with 1.5cm spacing | OPP, FDI, ADM, EDC, FDS | 45 degrees from sagittal plane | 110% OPP RMT |
|  | (Marconi, Koch et al. 2007) | N = 9 healthy controls | Landmark guided | At rest verified by EMG and during motor imagery | 7 x 7 grid with 1.5cm spacing | ADM, OPP, EDC, FCR | 45 degrees from sagittal plane | 110% RMT at which response measured in all muscles |
|  | (Marconi, Filippi et al. 2008) | N = 26 healthy controls | Landmark guided | At rest verified by EMG | 10.5 x 10.5cm with 1.5cm spacing | FCR, EDC, ADM | 45 degrees from sagittal plane | 120% RMT |
|  | (Massé‐Alarie, Bergin et al. 2017) | N = 14 healthy controls | Neuronavigated without MRI guidance | At rest and during 5% MVC verified by EMG | 1cm grid spacing | ECR, EDC, FCR,FDS | 45 degrees from sagittal plane | 120% RMT, 120% aMT @ 5% MVC wrist extension and grip |
|  | (Mathew, Kubler et al. 2016) | N = 11 young healthy controls | MRI guided neuronavigation | At rest verified by EMG | Grid with 0.3cm spacing | ECU, ECR, EDC | Not stated for during mapping | 110% ECU RMT |
|  | (McDonnell, Hillier et al. 2015) | N= 13 young healthy controls | Landmark navigated | At rest verified by EMG | Grid with 1cm spacing | APB | 45 degrees to sagittal plane | 110% APB RMT |
|  | (McGregor, Carpenter et al. 2012) | N= 7 young healthy controls N= 7 older healthy controls | MRI guided neuronavigation | Not stated | Grid with 1cm spacing | FDI | 45 degrees to sagittal plane | 110% FDI RMT |
|  | (McKay, Ridding et al. 2002) | N = 9 healthy controls | Landmark guided | Not stated, assumed at rest | Grid with 1cm spacing | FDI, APB, ADM | 45 degrees from sagittal plane | FDI RMT + 15% MSO |
|  | (McMillan, Watson et al. 1998) | N= 7 healthy controls | Landmark guided | Active verified by EMG | Grid with 1cm spacing | MM | Not explicitly stated, implied consistency with Brasil-Neto, 1992 (parallel to sagittal plane) | 10% above intensity to produce response >95% CI of mean EMG |
|  | (Meincke, Hewitt et al. 2016) | N= 11 healthy controls | MRI guided neuronavigation with robotic assistance | Not stated | 7 x 7 grid with 0.7cm spacing | FDI | 45 degrees to the sagittal plane | 120% FDI RMT |
|  | (Meincke, Hewitt et al. 2018) | N = 8 healthy controls | MRI guided neuronavigation with robotic assistance | At rest | 7 x 7 grid with 1cm spacing | FDI, PAM | 45 degrees to the sagittal plane | 120% RMT from 1st session for FDI, 140% FDI RMT for PAM (1^st^ session),  inflection point of FDI recruitment curve for PAM (2^nd^ session) |
|  | (Melgari, Pasqualetti et al. 2008) | N= 10 healthy controls | Landmark navigated | At rest | Spiral grid, 1cm intervals | ADM, FDI, OPP, APB, EDC, EIP, ECU, ERC, FDS, BB, TRI, DEL | 45 degrees to sagittal plane | 110% OPP RMT |
|  | (Meyer, Britton et al. 1991) | N = 20 healthy controls | Landmark navigated | 10% isometric contraction | Grid with 2cm spacing | ECR, FDI, BB, TA, QF, extensor digitorum brevis | Not stated, but appears to be varied between clockwise and counter clockwise | 150% RMT with 200mV threshold |
|  | (Miranda, de Carvalho et al. 1997) | N= 13 healthy controls | Navigation using digitized landmarks | At rest | Approximately 1cm spacing around responsive sites | ADM, FCR, BB | Parallel to sagittal plane and optimal orientation for MEP production | 120% ADM, FCR, BB RMT |
|  | (Mortifee, Stewart et al. 1994) | N= 6 young healthy controls | Landmark navigated | At rest | Grid with 1cm spacing | APB, ADM | Parallel to sagittal plane | 110% APB, ADM RMT |
|  | (Nakagawa, Takemi et al. 2020) | N = 12 healthy controls | Neuronavigated without MRI guidance | At rest | 10 x 8 grid with 1cm spacing | TA, Soleus, ED, RF | perpendicular to the saggital plane +/-20degrees | 120% RMT |
|  | (Ngomo, Mercier et al. 2013) | N = 15 healthy controls | MRI guided neuronavigation | Active during ~5% MVC | 1cm spacing | Infraspinatus | Not stated | 110% amT |
|  | (Ngomo, Leonard et al. 2012) | N= 12 young healthy controls | MRI guided neuronavigation | At rest and active verified by EMG | 7 x 8 grid with 1cm spacing | FDI | Not stated | 110% FDI RMT (rest), 110% FDI AMT (active) |
|  | (Ngomo, Leonard et al. 2012) | N = 11 young healthy controls | MRI guided neuronavigation | At rest or active | 8 x 8 grid with 1cm spacing | FDI | 45 degrees from sagittal plane | 110% RMT or AMT |
|  | (Nicolini, Harasym et al. 2019) | N = 37 healthy controls | Neuronavigated without MRI guidance | At rest | 6 x 5 grid with 1cm spacing, rotated to align with central sulcus | APB, FCR, BB | Not stated | 120% RMT for each muscle |
|  | (Niyazov, Butler et al. 2005) | N = 6 healthy controls | Landmark navigated | Not stated, assumed at rest | 1 x 2 cm grid | FDI | AP, PA or perpendicular to AP | 110% threshold |
|  | (Oliveri, Brighina et al. 1999) | N = 7 healthy controls | Landmark | At rest verified by EMG | 1cm lateral spacing from vertex, 2cm rostral / occipital | DEL, APB | Parallel to sagittal plane | 100% MSO |
|  | (Pascual-Leone, Nguyet et al. 1995) | N = 18  healthy controls | Landmark navigated | At rest verified by EMG | 5 x 5 grid with 1 cm spacing | Finger flexors and extensors | Parallel to the sagittal plane | 110% threshold |
|  | (Pitkanen, Kallioniemi et al. 2015) | N= 10 young healthy controls | MRI guided neuronavigation | At rest and active | Grid with 0.5cm spacing or no grid with approximated 1cm spacing | FDI, APB, ADM | Not stated | 1mV response at hotspot for FDI |
|  | (Pitkänen, Yazawa et al. 2019) | N = 10 healthy controls | Not stated | At rest, verified by EMG | Not stated | FDI, ECR | Perpendicular to the central sulcus | 105-110% RMT |
|  | (Plow, Varnerin et al. 2014) | N = 20 young healthy controls and N= 27 older healthy controls | Neuronavigated without MRI guidance | At rest | 0.3cm spacing in 8 radial directions from hotspot | BB | 8 radial directions from biceps hotspot | 110% BB RMT |
|  | (Plowman-Prine, Triggs et al. 2008) | N= 20 healthy controls | Landmark navigated | At rest | 7 x 7 grid with 1cm spacing | Suprahyoid muscle complex (throat), and pharyngeal activity | Parallel to sagittal plane | 115% throat, pharyngeal RMT |
|  | (Raffin, Pellegrino et al. 2015) | N= 13 healthy young controls | MRI guided neuronavigation | At rest and active | Along 45 degree to sagittal plane line, spaced 1cm apart, or roughly 1cm apart along the central sulcus | FDI, ADM | 45 degrees to the sagittal plane in line over central sulcus, 45 degrees to the sagittal plane tracing the central sulcus, and perpendicular to the central sulcus at all points | 120% of RMT of FDI for resting, and 120% of AMT for FDI for active |
|  | (Reijonen, Säisänen et al. 2020) | N = 10 healthy controls | MRI guided neuronavigation | Not stated, assumed at rest | A line along central sulcus with ~ 0.5cm spacing, and fixed 0.5cm gird | FDI, ADM, APB | Orientation at each test site producing largest response | 120% RMT, and 105% RMT |
|  | (Rödel, Laskawi et al. 2000) | N = 17 healthy controls | Not stated | Not stated | 1cm spacing along the interaural line from the vertex | APB, lower lip  (depressor anguli oris, depressor labii ingerioris) | Parallel to the sagittal plane | 120% RMT |
|  | (Rodel, Laskawi et al. 2001) | N = 16 healthy volunteers | Not stated | Not stated | 1cm spacing along the interaural line from the vertex | Frontalis muscles | Parallel to the sagittal plane | 120% RMT |
|  | (Rodel, Laskawi et al. 2003) | N = 17 healthy controls | Not stated | Active verified by EMG | 1cm lateral spacings from vertex along interaural line, and 2cm rostral / occipital to interauricular line | Lingual and lower lip | Parallel to sagittal plane | 120% RMT |
|  | (Rödel, Olthoff et al. 2004) | N = 6 healthy controls for each muscle group | Not stated | At rest verified by EMG | 1cm lateral spacings from vertex along interaural | Cricothyroid, vocalis | Parallel to sagittal plane | 120% RMT |
|  | (Roedel, Laskawi et al. 2001) | N = 17 healthy controls | Landmark guided | At rest | 1cm spacing along the interaural line, 2cm spacing along the rostral-occipital direction from the interauricular line | OO | Parallel to the sagittal plane | 120% RMT |
|  | (Röricht, Machetanz et al. 2001) | N = 10 healthy controls | Assume landmark guided | At rest | 1 x 2 cm grid | FDI | Parallel to sagittal plane | 150% RMT |
|  | (Rossi, Pasqualetti et al. 1998) | N = 10 healthy controls | Landmark guided | At rest verified by EMG | 1cm spacing | FDI, ADM | Perpendicular to the central sulcus | 110% RMT of each muscle |
|  | (Säisänen, Julkunen et al. 2015) | N = 8 healthy controls | MRI guided neuronavigation | At rest verified by EMG | Grid with 0.5cm spacing | MM, OO, masseter, frontalis | 45 degrees from sagittal plane, and parallel to the sagittal plane | 110% mentalis |
|  | (Sankarasubramanian, Roelle et al. 2015) | N = 20 young healthy controls | Neuronavigated without MRI guidance | At rest | 0.3cm in 8 radial directions until no response found | BB | 45 degrees from sagittal plane | 110% RMT (10µV response threshold) |
|  | (Schabrun and Ridding 2007) | N = 24 healthy controls | Landmark navigated | At rest verified by EMG | 1cm x 1cm grid | FDI, ADM, APB | 45 degrees from sagittal plane | 120% RMT of each muscle |
|  | (Schabrun, Stinear et al. 2008) | N = 10 healthy controls | Landmark guided | At rest verified by EMG | Grid with 1cm spacing | FDI, APB, ADM | 45 degrees from sagittal plane | 120% RMT of each muscle |
|  | (Schulze-Bonhage, Cichon et al. 1998) | N = 21 young healthy controls | Landmark guided | Active state | 2cm spacing centered around Cz | FDI, DEL | Parallel to sagittal plane | 100% MSO for ipsilateral responses, 115% RMT for contralateral |
|  | (Singh, Hamdy et al. 1997) | N = 3 healthy controls | MRI guided neuronavigation | Not stated, assumed at rest | 14 cm x 10cm with 2cm spacing in anteropostero direction and 1cm in mediolateral | APB | 45 degrees from sagittal plane | 120% RMT |
|  | (Sinitsyn, Chernyavskiy et al. 2019) | N = 8 young healthy controls | MRI guided neuronavigation | Not stated, assumed at rest | 7 x 7 square grid of 0.763cm at peeling depth 20mm | APB | Perpendicular to the central sulcus | 110% RMT |
|  | (Smith and Fisher 2018) | N = 12 older (>65) and N = 13 young healthy controls | Neuronavigation without MRI guidance to match landmarks | Active verified by EMG | 6 x 4 grid with 1cm spacing | EO, LL, GMED | Not stated | 120% AMT |
|  | (Sparing, Buelte et al. 2008) | N= 10 young healthy controls | Landmark guided, function guided, or MRI guided | At rest | 7.5cm x 6cm grid with 1.5cm spacing | FDI | 45 degrees to sagittal plane | To produce 0.5-1.0mV response |
|  | (Stephani, Paulus et al. 2016) | N = 11 healthy controls | Landmark navigated | At rest | Hexagonal grid centered around hotspot of FDI with 0.57cm spacing between points | FDI, ADM, ECR, BB | 45 degrees from sagittal plane | 1mV in FDI |
|  | (Suzuki, Kirimoto et al. 2012) | N = 10 young healthy controls | Landmark navigated | At rest | 9 x 9 grid with 1.5cm spacing | ECR, FCR | 45 degrees from sagittal plane | 120% RMT for ECR, FCR |
|  | (Svensson, Romaniello et al. 2003) | N = 11 young healthy controls | Landmark navigated | At rest verified by EMG | Grid with 1 cm spacing | Tongue dorsum, FDI | 45 degrees from sagittal plane | 120% Threshold |
|  | (Thickbroom, Byrnes et al. 2005) | N = 20 | Landmark navigated | Active, at ~10% of maximum verified by EMG | 1cm latitude x 2cm longitude | FDI | Not stated | RMT + 20% MSO |
|  | (Thickbroom, Sammut et al. 1998) | N = 5 healthy controls | Landmark navigated | Active, at ~10% of maximum verified by EMG | 6 grid sites | FDI | Parallel to the sagittal plane | Increasing by 5% MSO from 10% below FDI motor threshold until responses saturate |
|  | (Thickbroom, Byrnes et al. 1999) | N = 5 healthy controls | Landmark navigated | Active, at ~10% of maximum verified by EMG | Grid with 1cm “latitude” and 2cm “longitude” spacing | APB | Not stated. | 120% above motor threshold |
|  | (Thompson, Thickbroom et al. 1997) | N = 15 | Landmark navigated | Active, at ~10% of maximum verified by EMG | 1cm latitude x 2cm longitude | SCM (bilateral), APB, | Parallel to the sagittal plane | 100% MSO in SCM, 120% RMT in APB |
|  | (Triggs, Subramanium et al. 1999) | N = 9 | Landmark navigated | At rest verified by audio feedback | 9 x 9 cm^2 grid | APB, FCR | Parallel to the sagittal plane | 120% RMT APB |
|  | (Tsao, Danneels et al. 2011) | N = 11 | Landmark navigated | ~20% Maximum RMS EMG | 5 x 7 grid with 1cm spacing | Erector spinae (longissiumus), lumbar multifidus | Parallel to the sagittal plane | 100% MSO |
|  | (Tsao, Galea et al. 2008) | N = 11 young healthy controls | Landmark navigated | 10% MVC | 5 x 5 cm grid | Transversus abdominus | 45 degrees from sagittal plane | 120% AMT |
|  | (Turnbull, Hamdy et al. 1999) | N = 9 healthy controls | Landmark navigated | At rest | 12 x 8cm grid with 2 cm spacing in anteropostero direction, 1cm mediolateral spacing | Anal, rectal and TA | 45 degrees from sagittal plane | 20% stimulator output above threshold eliciting responses in anal, rectal and TA |
|  | (Uy, Ridding et al. 2002) | N= 8 healthy controls | Landmark navigated | At rest | Grid with 1cm spacing | FDI, APB, ADM | 45 degrees to sagittal plane | 115% FDI, APB, ADM RMT |
|  | (Van De Ruit, Perenboom et al. 2015) | N= 7 young healthy controls, N=4 young healthy controls | MRI guided neuronavigation | At rest | Randomly stimulating within a 6cm x 6cm square, or at each point within a 7 x 7 grid with 1cm spacing | FDI | 45 degrees to sagittal plane | 120% FDI RMT |
|  | (van de Ruit and Grey 2017) | N = 44 healthy young controls | Neuronavigated | At rest – verified by EMG | Randomly stimulating within a 6cm x 6cm square | EDC | 45 degrees to sagittal plane | 120% RMT EDC |
|  | (van de Ruit and Grey 2019) | N = 40 healthy young controls | Neuronavigated | At rest – verified by EMG | Randomly stimulating within a 6cm x 6cm square | BB or FDI | 45 degrees to sagittal plane | 120% RMT of BB or FDI |
|  | (Ward, Bryant et al. 2016) | N = 15 healthy controls | Landmark navigated | During isometric quadriceps contraction at 10% of body weight | Grid with 1 cm spacing | Rectus femoris | Parallel to the sagittal plane | 120% of the rectus femoris |
|  | (Wassermann, Wang et al. 1996) | N = 4 | Landmark guided | Not stated, assumed at rest | 7 x 7 grid with 1 cm spacing | FDI | Parallel to the sagittal plane | 110% RMT |
|  | (Weiss, Nettekoven et al. 2013) | N= 10 healthy young controls | MRI guided neuronavigation | At rest | Grid with 0.5cm spacing | APB, PM, MM, LT | Perpendicular to central sulcus for hand musculature, perpendicular to course of interhemispheric fissure for foot | 110% APB, PM, MM, LT RMT |
|  | (Wilson, Thickbroom et al. 1995) | N = 5 healthy controls | Landmark guided | at rest and at ~10% MVC | 1cm spacing | APB | parallel to sagittal plane | RMT + 20% MSO |
|  | (Wilson, Thickbroom et al. 1993) | N = 16 healthy controls | Landmark guided | Active verified by EMG | Grid with 1cm spacing | APB, ADM | Parallel to sagittal plane | 120% APB AMT |
|  | (Wolf, Butler et al. 2004) | N= 9 healthy males | Landmark navigated | At rest and active | Grid with 1cm spacing | EDC | Parallel to sagittal plane | 110% EDC RMT |
|  | (Zanette, Tinazzi et al. 1997) | N= 10 healthy controls | Landmark navigated | At rest (active contraction not measured in control group) | Grid with 1cm spacing | APB, FCR, BB, DEL | Parallel to sagittal plane | 110% of highest RMT for all muscles |
|  | (Zdunczyk, Fleischmann et al. 2013) | N= 10 healthy controls | MRI guided neuronavigation | At rest verified by EMG | Grid with 0.5cm spacing | FDI | Orthogonal to gyri or one of the principle directions, randomly selected | 110% FDI RMT |
|  | (Ziemann, Wittenberg et al. 2002) | N = 6 healthy controls | Landmark navigated | At rest | Grid with 1cm spacing | BB, APB, OO, TA | 45 degrees from sagittal plane | 120% RMT |
| *young = 18-35, EMG = electromyography, RMT = resting motor threshold (50µV threshold), AMT = active motor threshold, CMEP = cervico-medullary evoked potential, MSO = maximum stimulator output,  FDI = first dorsal interosseus, ADM = abductor digiti minimi, AH = abductor hallucis, APB = abductor pollicis brevis, BB = biceps brachii, EDC = extensor digitorum communis, ECU = extensor carpi unlaris, ECR = extensor capri radialis, TA = tibialis anterior, PM = plantaris muscle, EO = external oblique, LL = thoracolumbar longissimus pars lumorum, GMED = gluteus medius, MM = mentalis muscle, LT = lateral tongue, PAM = posterior auricular muscle, TRI = triceps, OPP = opponens pollicis, EIP = extensor indicus proprius, DEL = deltoid, FDS = flexor digitorum superficialis, FCR = flexor carpi radialis,,EO = external oblique, OO = orbicularis oculi, QF = quadriceps femoris | | | | | | | | |
|  | | | | | | | | |

Ahdab, R., S. S. Ayache, P. Brugières, W. H. Farhat and J. P. Lefaucheur (2016). "The Hand Motor Hotspot is not Always Located in the Hand Knob: A Neuronavigated Transcranial Magnetic Stimulation Study." Brain Topogr **29**(4): 590-597.

Ahdab, R., S. S. Ayache, W. H. Farhat, V. Mylius, S. Schmidt, P. Brugieres and J. P. Lefaucheur (2014). "Reappraisal of the anatomical landmarks of motor and premotor cortical regions for image-guided brain navigation in TMS practice." Human Brain Mapping **35**(5): 2435-2447.

Aziz, Q., J. C. Rothwell, S. Hamdy, J. Barlow and D. G. Thompson (1996). "The topographic representation of esophageal motor function on the human cerebral cortex." Gastroenterology **111**(4): 855-862.

Bashir, S., J. M. Perez, J. C. Horvath and A. Pascual-Leone (2013). "Differentiation of motor cortical representation of hand muscles by navigated mapping of optimal TMS current directions in healthy subjects." Journal of Clinical Neurophysiology **30**(4): 390-395.

Bastings, E. P., H. D. Gage, J. P. Greenberg, G. Hammond, L. Hernandez, P. Santago, C. A. Hamilton, D. M. Moody, K. D. Singh and P. E. Ricci (1998). "Co-registration of cortical magnetic stimulation and functional magnetic resonance imaging." Neuroreport **9**(9): 1941-1946.

Bastings, E. P., J. P. Greenberg and D. C. Good (2002). "Hand motor recovery after stroke: a transcranial magnetic stimulation mapping study of motor output areas and their relation to functional status." Neurorehabilitation and Neural Repair **16**(3): 275-282.

Bernard, J. A. and R. D. Seidler (2012). "Evidence for motor cortex dedifferentiation in older adults." Neurobiol Aging **33**(9): 1890-1899.

Bernard, J. A. and R. D. Seidler (2012). "Hand dominance and age have interactive effects on motor cortical representations." PloS one **7**(9): e45443.

Boroojerdi, B., H. Foltys, T. Krings, U. Spetzger, A. Thron and R. Töpper (1999). "Localization of the motor hand area using transcranial magnetic stimulation and functional magnetic resonance imaging." Clinical Neurophysiology **110**(4): 699-704.

Brasil-Neto, J. P., L. M. McShane, P. Fuhr, M. Hallett and L. G. Cohen (1992). "Topographic mapping of the human motor cortex with magnetic stimulation: factors affecting accuracy and reproducibility." Electroencephalography and Clinical Neurophysiology/ Evoked Potentials **85**(1): 9-16.

Brogårdh, C., F. W. Johansson, F. Nygren and B. H. Sjölund (2010). "Mode of hand training determines cortical reorganisation: a randomized controlled study in healthy adults." Journal of rehabilitation medicine **42**(8): 789-794.

Brouwer, B. and D. H. Hopkins-Rosseel (1997). "Motor cortical mapping of proximal upper extremity muscles following spinal cord injury." Spinal Cord **35**(4): 205.

Buick, A. R., N. C. Kennedy and R. G. Carson (2016). "Characteristics of corticospinal projections to the intrinsic hand muscles in skilled harpists." Neuroscience letters **612**: 87-91.

Cavaleri, R., L. S. Chipchase, H. Massé‐Alarie, S. M. Schabrun, M. A. Shraim and P. W. Hodges (2020). "Corticomotor reorganization during short‐term visuomotor training in the lower back: A randomized controlled study." Brain and Behavior **10**(8): e01702.

Cavaleri, R., S. M. Schabrun and L. S. Chipchase (2018). "The reliability and validity of rapid transcranial magnetic stimulation mapping." Brain Stimulation **11**(6): 1291-1295.

Chernyavskiy, A. Y., D. O. Sinitsyn, A. G. Poydasheva, I. S. Bakulin, N. A. Suponeva and M. A. Piradov (2019). "Accuracy of Estimating the Area of Cortical Muscle Representations from TMS Mapping Data using Voronoi Diagrams." Brain topography: 1-14.

Chieffo, R., L. Straffi, A. Inuggi, J. J. Gonzalez-Rosa, F. Spagnolo, E. Coppi, A. Nuara, E. Houdayer, G. Comi and L. Leocani (2016). "Motor Cortical Plasticity to Training Started in Childhood: The Example of Piano Players." PLOS ONE **11**(6): e0157952.

Cicinelli, P., R. Traversa, A. Bassi, G. Scivoletto and P. M. Rossini (1997). "Interhemispheric differences of hand muscle representation in human motor cortex." Muscle and Nerve **20**(5): 535-542.

Cicinelli, P., R. Traversa and P. Rossini (1997). "Post-stroke reorganization of brain motor output to the hand: a 2–4 month follow-up with focal magnetic transcranial stimulation." Electroencephalography and Clinical Neurophysiology/Electromyography and Motor Control **105**(6): 438-450.

Classen, J., U. Knorr, K. J. Werhahn, G. Schlaug, E. Kunesch, L. G. Cohen, R. J. Seitz and R. Benecke (1998). "Multimodal output mapping of human central motor representation on different spatial scales." Journal of Physiology **512**(1): 163-179.

Corneal, S. F., A. J. Butler and S. L. Wolf (2005). "Intra- and intersubject reliability of abductor pollicis brevis muscle motor map characteristics with transcranial magnetic stimulation." Archives of Physical Medicine and Rehabilitation **86**(8): 1670-1675.

Davies, J. L. (2020). "Using transcranial magnetic stimulation to map the cortical representation of lower-limb muscles." Clinical Neurophysiology Practice.

de Goede, A. A., E. M. ter Braack and M. J. A. M. van Putten (2018). "Accurate Coil Positioning is Important for Single and Paired Pulse TMS on the Subject Level." Brain Topography **31**(6): 917-930.

Delvaux, V., G. Alagona, P. Gérard, V. De Pasqua, G. Pennisi and A. M. de Noordhout (2003). "Post-stroke reorganization of hand motor area: a 1-year prospective follow-up with focal transcranial magnetic stimulation." Clinical Neurophysiology **114**(7): 1217-1225.

Devanne, H., F. Cassim, C. Ethier, L. Brizzi, A. Thevenon and C. Capaday (2006). "The comparable size and overlapping nature of upper limb distal and proximal muscle representations in the human motor cortex." European Journal of Neuroscience **23**(9): 2467-2476.

Elgueta-Cancino, E., S. Schabrun and P. Hodges (2018). "Is the organization of the primary motor cortex in low back pain related to pain, movement, and/or sensation?" The Clinical journal of pain **34**(3): 207-216.

Engelhardt, M. and T. Picht (2020). "1 Hz Repetitive Transcranial Magnetic Stimulation of the Primary Motor Cortex: Impact on Excitability and Task Performance in Healthy Subjects." Journal of Neurological Surgery Part A: Central European Neurosurgery **81**(02): 147-154.

Ferbert, A., D. Caramia, A. Priori, L. Bertolasi and J. Rothwell (1992). "Cortical projection to erector spinae muscles in man as assessed by focal transcranial magnetic stimulation." Electroencephalography and Clinical Neurophysiology/Evoked Potentials Section **85**(6): 382-387.

Forner-Cordero, A., M. Steyvers, O. Levin, K. Alaerts and S. P. Swinnen (2008). "Changes in corticomotor excitability following prolonged muscle tendon vibration." Behavioural brain research **190**(1): 41-49.

Forster, M. T., M. Limbart, V. Seifert and C. Senft (2014). "Test-retest reliability of navigated transcranial magnetic stimulation of the motor cortex." Neurosurgery **10 Suppl 1**: 51-55; discussion 55-56.

Forster, M. T., C. Senft, E. Hattingen, M. Lorei, V. Seifert and A. Szelenyi (2012). "Motor cortex evaluation by ntms after surgery of central region tumors: A feasibility study." Acta Neurochirurgica **154**(8): 1351-1359.

Freund, P., J. Rothwell, M. Craggs, A. J. Thompson and S. Bestmann (2011). "Corticomotor representation to a human forearm muscle changes following cervical spinal cord injury." European Journal of Neuroscience **34**(11): 1839-1846.

Gallas, S., J. Marie, A. Leroi and E. Verin (2009). "Impact of swallowing and ventilation on oropharyngeal cortical representation." Respiratory physiology & neurobiology **167**(2): 208-213.

Ginhoux, R., P. Renaud, L. Zorn, L. Goffin, B. Bayle, J. Foucher, J. Lamy, J. P. Armspach and M. de Mathelin (2013). "A custom robot for Transcranial Magnetic Stimulation: first assessment on healthy subjects." Conference proceedings : .. **Annual International Conference of the IEEE Engineering in Medicine and Biology Society. IEEE Engineering in Medicine and Biology Society. Annual Conference. 2013**: 5352-5355.

Green, A., P. W. Cheong, S. Fook-Chong, R. Tiruchelvarayan, C. M. Guo, W. M. Yue, J. Chen and Y. L. Lo (2015). "Cortical reorganization is associated with surgical decompression of cervical spondylotic myelopathy." Neural plasticity **2015**.

Guerra, A., S. Petrichella, L. Vollero, D. Ponzo, P. Pasqualetti, S. Määttä, E. Mervaala, M. Könönen, F. Bressi and G. Iannello (2015). "Neurophysiological features of motor cortex excitability and plasticity in subcortical ischemic vascular dementia: a TMS mapping study." Clinical Neurophysiology **126**(5): 906-913.

Hamdy, S., Q. Aziz, J. C. Rothwell, K. D. Singh, J. Barlow, D. G. Hughes, R. C. Tallis and D. G. Thompson (1996). "The cortical topography of human swallowing musculature in health and disease." Nature medicine **2**(11): 1217.

Herwig, U., K. Kölbel, A. P. Wunderlich, A. Thielscher, C. von Tiesenhausen, M. Spitzer and C. Schönfeldt-Lecuona (2002). "Spatial congruence of neuronavigated transcranial magnetic stimulation and functional neuroimaging." Clinical Neurophysiology **113**(4): 462-468.

Inuggi, A., M. Filippi, R. Chieffo, F. Agosta, M. A. Rocca, J. J. González-Rosa, M. Cursi, G. Comi and L. Leocani (2010). "Motor area localization using fMRI-constrained cortical current density reconstruction of movement-related cortical potentials, a comparison with fMRI and TMS mapping." Brain research **1308**: 68-78.

Jonker, Z. D., R. van der Vliet, C. M. Hauwert, C. Gaiser, J. H. M. Tulen, J. N. van der Geest, O. Donchin, G. M. Ribbers, M. A. Frens and R. W. Selles (2019). "TMS motor mapping: Comparing the absolute reliability of digital reconstruction methods to the golden standard." Brain Stimulation **12**(2): 309-313.

Jono, Y., Y. Chujo, Y. Nomura, K. Tani, Y. Nikaido, R. Hatanaka and K. Hiraoka (2015). "The effect of tonic contraction of the finger muscle on the motor cortical representation of the contracting adjacent muscle." Somatosensory & motor research **32**(2): 114-121.

Julkunen, P. (2014). "Methods for estimating cortical motor representation size and location in navigated transcranial magnetic stimulation." Journal of neuroscience methods **232**: 125-133.

Julkunen, P., L. Saisanen, N. Danner, E. Niskanen, T. Hukkanen, E. Mervaala and M. Kononen (2009). "Comparison of navigated and non-navigated transcranial magnetic stimulation for motor cortex mapping, motor threshold and motor evoked potentials." Neuroimage **44**(3): 790-795.

Kallioniemi, E. and P. Julkunen (2016). "Alternative Stimulation Intensities for Mapping Cortical Motor Area with Navigated TMS." Brain Topography **29**(3): 395-404.

Kallioniemi, E., M. Pitkänen, M. Könönen, R. Vanninen and P. Julkunen (2016). "Localization of cortical primary motor area of the hand using navigated transcranial magnetic stimulation, BOLD and arterial spin labeling fMRI." Journal of Neuroscience Methods **273**: 138-148.

Kallioniemi, E., M. Pitkanen, L. Saisanen and P. Julkunen (2015). "Onset latency of motor evoked potentials in motor cortical mapping with neuronavigated transcranial magnetic stimulation." Open Neurology Journal **9**(1): 62-69.

Kantelhardt, S. R., T. Fadini, M. Finke, K. Kallenberg, J. Siemerkus, V. Bockermann, L. Matthaeus, W. Paulus, A. Schweikard, V. Rohde and A. Giese (2010). "Robot-assisted image-guided transcranial magnetic stimulation for somatotopic mapping of the motor cortex: A clinical pilot study." Acta Neurochirurgica **152**(2): 333-343.

Komoda, Y., T. Iida, M. Kothari, O. Komiyama, L. Baad-Hansen, M. Kawara, B. Sessle and P. Svensson (2015). "Repeated tongue lift movement induces neuroplasticity in corticomotor control of tongue and jaw muscles in humans." Brain research **1627**: 70-79.

Kosik, K. B., M. Terada, C. P. Drinkard, R. S. Mccann and P. A. Gribble (2017). "Potential Corticomotor Plasticity in Those with and without Chronic Ankle Instability." Medicine and science in sports and exercise **49**(1): 141-149.

Kothari, M., P. Svensson, J. Jensen, A. Kjærsgaard, K. Jeonghee, J. F. Nielsen, M. Ghovanloo and L. Baad-Hansen (2013). "Training-induced cortical plasticity compared between three tongue-training paradigms." Neuroscience **246**: 1-12.

Kraus, D. and A. Gharabaghi (2015). "Projecting navigated TMS sites on the gyral anatomy decreases inter-subject variability of cortical motor maps." Brain Stimulation **8**(4): 831-837.

Kraus, D. and A. Gharabaghi (2016). "Neuromuscular Plasticity: Disentangling Stable and Variable Motor Maps in the Human Sensorimotor Cortex." Neural Plasticity **2016**.

Krause, P., S. Förderreuther and A. Straube (2006). "TMS motor cortical brain mapping in patients with complex regional pain syndrome type I." Clinical Neurophysiology **117**(1): 169-176.

Krings, T., B. Buchbinder, W. Butler, K. Chiappa, H. Jiang, G. Cosgrove and B. Rosen (1997). "Functional magnetic resonance imaging and transcranial magnetic stimulation: complementary approaches in the evaluation of cortical motor function." Neurology **48**(5): 1406-1416.

Krings, T., C. Naujokat and D. G. v. Keyserlingk (1998). "Representation of cortical motor function as revealed by stereotactic transcranial magnetic stimulation." Electroencephalography and Clinical Neurophysiology/Electromyography and Motor Control **109**(2): 85-93.

Labyt, E., E. Houdayer, F. Cassim, J. Bourriez, P. Derambure and H. Devanne (2007). "Motor representation areas in epileptic patients with focal motor seizures: a TMS study." Epilepsy research **75**(2-3): 197-205.

Lewis, G. N. and W. D. Byblow (2004). "The effects of repetitive proprioceptive stimulation on corticomotor representation in intact and hemiplegic individuals." Clinical neurophysiology **115**(4): 765-773.

Lewis, G. N., N. Signal and D. Taylor (2014). "Reliability of lower limb motor evoked potentials in stroke and healthy populations: how many responses are needed?" Clinical Neurophysiology **125**(4): 748-754.

Li, W., X. Li, T. Lin, Y. Jing, C. Wu, M. Li, Y. Lan and G. Xu (2020). "TMS brain mapping of the pharyngeal cortical representation in healthy subjects." Brain Stimulation.

Littmann, A. E., C. L. McHenry and R. K. Shields (2013). "Variability of motor cortical excitability using a novel mapping procedure." J Neurosci Methods **214**(2): 137-143.

Lu, S., L. Baad-Hansen, Z. Zhang and P. Svensson (2013). "One hour jaw muscle training does not evoke plasticity in the corticomotor control of the masseter muscle." Archives of oral biology **58**(10): 1483-1490.

Lucente, G., S. Lam, H. Schneider and T. Picht (2017). "Preservation of motor maps with increased motor evoked potential amplitude threshold in RMT determination." Acta Neurochirurgica: 1-6.

Malcolm, M. P., W. J. Triggs, K. E. Light, O. Shechtman, G. Khandekar and L. J. Gonzalez Rothi (2006). "Reliability of motor cortex transcranial magnetic stimulation in four muscle representations." Clinical Neurophysiology **117**(5): 1037-1046.

Marconi, B., G. M. Filippi, G. Koch, C. Pecchioli, S. Salerno, R. Don, F. Camerota, V. M. Saraceni and C. Caltagirone (2008). "Long-term effects on motor cortical excitability induced by repeated muscle vibration during contraction in healthy subjects." Journal of the neurological sciences **275**(1-2): 51-59.

Marconi, B., G. Koch, C. Pecchioli, P. Cavallari and C. Caltagirone (2007). "Breakdown of inhibitory effects induced by foot motor imagery on hand motor area in lower-limb amputees." Clinical neurophysiology **118**(11): 2468-2478.

Marconi, B., C. Pecchioli, G. Koch and C. Caltagirone (2007). "Functional overlap between hand and forearm motor cortical representations during motor cognitive tasks." Clinical neurophysiology **118**(8): 1767-1775.

Massé‐Alarie, H., M. J. Bergin, C. Schneider, S. Schabrun and P. W. Hodges (2017). "“Discrete peaks” of excitability and map overlap reveal task‐specific organization of primary motor cortex for control of human forearm muscles." Human brain mapping **38**(12): 6118-6132.

Mathew, J., A. Kubler, R. Bauer and A. Gharabaghi (2016). "Probing Corticospinal Recruitment Patterns and Functional Synergies with Transcranial Magnetic Stimulation." Frontiers in Cellular Neuroscience **10**: 175.

McDonnell, M. N., S. L. Hillier, G. M. Opie, M. Nowosilskyj, M. Haberfield and G. Todd (2015). "Continuous passive movement does not influence motor maps in healthy adults." Frontiers in Human Neuroscience **9**.

McGregor, K. M., H. Carpenter, E. Kleim, A. Sudhyadhom, K. D. White, A. J. Butler, J. Kleim and B. Crosson (2012). "Motor map reliability and aging: A TMS/fMRI study." Experimental Brain Research **219**(1): 97-106.

McKay, D. R., M. C. Ridding, P. D. Thompson and T. S. Miles (2002). "Induction of persistent changes in the organisation of the human motor cortex." Experimental brain research **143**(3): 342-349.

McMillan, A. S., C. Watson and D. Walshaw (1998). "Transcranial magnetic-stimulation mapping of the cortical topography of the human masseter muscle." Archives of oral biology **43**(12): 925-931.

Meincke, J., M. Hewitt, G. Batsikadze and D. Liebetanz (2016). "Automated TMS hotspot-hunting using a closed loop threshold-based algorithm." NeuroImage **124**: 509-517.

Meincke, J., M. Hewitt, M. Reischl, R. Rupp, C. Schmidt-Samoa and D. Liebetanz (2018). "Cortical representation of auricular muscles in humans: A robot-controlled TMS mapping and fMRI study." PLOS ONE **13**(7): e0201277.

Melgari, J. M., P. Pasqualetti, F. Pauri and P. M. Rossini (2008). "Muscles in "Concert": Study of primary motor cortex upper limb functional topography." PLoS ONE **3 (8) (no pagination)**(e3069).

Meyer, B.-U., T. Britton, H. Kloten, H. Steinmetz and R. Benecke (1991). "Coil placement in magnetic brain stimulation related to skull and brain anatomy." Electroencephalography and Clinical Neurophysiology/Evoked Potentials Section **81**(1): 38-46.

Miranda, P. C., M. de Carvalho, I. Conceicao, M. L. Luis and E. Ducla-Soares (1997). "A new method for reproducible coil positioning in transcranial magnetic stimulation mapping." Electroencephalography & Clinical Neurophysiology **105**(2): 116-123.

Mortifee, P., H. Stewart, M. Schulzer and A. Eisen (1994). "Reliability of transcranial magnetic stimulation for mapping the human motor cortex." Electroencephalography and Clinical Neurophysiology/ Evoked Potentials **93**(2): 131-137.

Nakagawa, K., M. Takemi, T. Nakanishi, A. Sasaki and K. Nakazawa (2020). "Cortical reorganization of lower-limb motor representations in an elite archery athlete with congenital amputation of both arms." NeuroImage: Clinical **25**: 102144.

Ngomo, S., G. Leonard and C. Mercier (2012). "Influence of the amount of use on hand motor cortex representation: effects of immobilization and motor training." Neuroscience **220**: 208-214.

Ngomo, S., G. Leonard, H. Moffet and C. Mercier (2012). "Comparison of transcranial magnetic stimulation measures obtained at rest and under active conditions and their reliability." Journal of neuroscience methods **205**(1): 65-71.

Ngomo, S., C. Mercier and J.-S. Roy (2013). "Cortical mapping of the infraspinatus muscle in healthy individuals." BMC neuroscience **14**(1): 1-7.

Nicolini, C., D. Harasym, C. V. Turco and A. J. Nelson (2019). "Human motor cortical organization is influenced by handedness." Cortex **115**: 172-183.

Niyazov, D., A. Butler, Y. Kadah, C. Epstein and X. Hu (2005). "Functional magnetic resonance imaging and transcranial magnetic stimulation: effects of motor imagery, movement and coil orientation." Clinical Neurophysiology **116**(7): 1601-1610.

Oliveri, M., F. Brighina, V. La Bua, D. Buffa, A. Aloisio and B. Fierro (1999). "Reorganization of cortical motor area in prior polio patients." Clinical Neurophysiology **110**(5): 806-812.

Pascual-Leone, A., D. Nguyet, L. G. Cohen, J. P. Brasil-Neto, A. Cammarota and M. Hallett (1995). "Modulation of muscle responses evoked by transcranial magnetic stimulation during the acquisition of new fine motor skills." Journal of neurophysiology **74**(3): 1037-1045.

Pitkanen, M., E. Kallioniemi and P. Julkunen (2015). "Extent and Location of the Excitatory and Inhibitory Cortical Hand Representation Maps: A Navigated Transcranial Magnetic Stimulation Study." Brain Topography **28**(5): 657-665.

Pitkänen, M., S. Yazawa, K. Airaksinen, P. Lioumis, J. Nurminen, E. Pekkonen and J. P. Mäkelä (2019). "Localization of Sensorimotor Cortex Using Navigated Transcranial Magnetic Stimulation and Magnetoencephalography." Brain topography: 1-9.

Plow, E. B., N. Varnerin, D. A. Cunningham, D. Janini, C. Bonnett, A. Wyant, J. Hou, V. Siemionow, X. F. Wang, A. G. Machado and G. H. Yue (2014). "Age-related weakness of proximal muscle studied with motor cortical mapping: a TMS study." PLoS ONE [Electronic Resource] **9**(2): e89371.

Plowman-Prine, E. K., W. J. Triggs, M. P. Malcolm and J. C. Rosenbek (2008). "Reliability of transcranial magnetic stimulation for mapping swallowing musculature in the human motor cortex." Clinical Neurophysiology **119**(10): 2298-2303.

Raffin, E., G. Pellegrino, V. Di Lazzaro, A. Thielscher and H. R. Siebner (2015). "Bringing transcranial mapping into shape: Sulcus-aligned mapping captures motor somatotopy in human primary motor hand area." NeuroImage **120**: 164-175.

Reijonen, J., L. Säisänen, M. Könönen, A. Mohammadi and P. Julkunen (2020). "The effect of coil placement and orientation on the assessment of focal excitability in motor mapping with navigated transcranial magnetic stimulation." Journal of Neuroscience Methods **331**: 108521.

Rödel, R., R. Laskawi and H. Markus (2000). "Motor potentials of lower-lip mimetic muscles and distal arm muscles to cortical transcranial magnetic stimulation: The possibility of one-dimensional separation of two cortical representation areas." ORL **62**(2): 96-99.

Rodel, R., R. Laskawi, H. Markus and K. Wenke (2001). "Cortical transcranial magnetic stimulation of the frontalis muscle: characteristic features and details on motor-evoked potentials of the forehead." ORL J Otorhinolaryngol Relat Spec **63**(2): 66-71.

Rodel, R. M., R. Laskawi and H. Markus (2003). "Tongue representation in the lateral cortical motor region of the human brain as assessed by transcranial magnetic stimulation." Annals of Otology, Rhinology & Laryngology **112**(1): 71-76.

Rödel, R. M., A. Olthoff, F. Tergau, K. Simonyan, D. Kraemer, H. Markus and E. Kruse (2004). "Human cortical motor representation of the larynx as assessed by transcranial magnetic stimulation (TMS)." The Laryngoscope **114**(5): 918-922.

Roedel, R. M., R. Laskawi and H. Markus (2001). "Cortical representation of the orbicularis oculi muscle as assessed by transcranial magnetic stimulation (TMS)." The Laryngoscope **111**(11): 2005-2011.

Röricht, S., J. Machetanz, K. Irlbacher, L. Niehaus, E. Biemer and B. U. Meyer (2001). "Reorganization of human motor cortex after hand replantation." Annals of neurology **50**(2): 240-249.

Rossi, S., P. Pasqualetti, F. Tecchio, A. Sabato and P. M. Rossini (1998). "Modulation of corticospinal output to human hand muscles following deprivation of sensory feedback." Neuroimage **8**(2): 163-175.

Säisänen, L., P. Julkunen, S. Kemppainen, N. Danner, A. Immonen, E. Mervaala, S. Määttä, A. Muraja-Murro and M. Könönen (2015). "Locating and outlining the cortical motor representation areas of facial muscles with navigated transcranial magnetic stimulation." Neurosurgery **77**(3): 394-405.

Sankarasubramanian, V., S. M. Roelle, C. E. Bonnett, D. Janini, N. M. Varnerin, D. A. Cunningham, J. S. Sharma, K. A. Potter-Baker, X. Wang, G. H. Yue and E. B. Plow (2015). "Reproducibility of transcranial magnetic stimulation metrics in the study of proximal upper limb muscles." Journal of electromyography and kinesiology : official journal of the International Society of Electrophysiological Kinesiology **25**(5): 754-764.

Schabrun, S. M. and M. C. Ridding (2007). "The influence of correlated afferent input on motor cortical representations in humans." Experimental Brain Research **183**(1): 41-49.

Schabrun, S. M., C. M. Stinear, W. D. Byblow and M. C. Ridding (2008). "Normalizing motor cortex representations in focal hand dystonia." Cerebral cortex **19**(9): 1968-1977.

Schulze-Bonhage, A., B. Cichon and A. Ferbert (1998). "Cortical representation of proximal and distal arm muscles as assessed by focal transcranial magnetic stimulation." Electromyography and clinical neurophysiology **38**(2): 81-86.

Singh, K. D., S. Hamdy, Q. Aziz and D. Thompson (1997). "Topographic mapping of trans-cranial magnetic stimulation data on surface rendered MR images of the brain." Electroencephalography and Clinical Neurophysiology/Electromyography and Motor Control **105**(5): 345-351.

Sinitsyn, D. O., A. Y. Chernyavskiy, A. G. Poydasheva, I. S. Bakulin, N. A. Suponeva and M. A. Piradov (2019). "Optimization of the Navigated TMS Mapping Algorithm for Accurate Estimation of Cortical Muscle Representation Characteristics." Brain sciences **9**(4): 88.

Smith, J. A. and B. E. Fisher (2018). "Anticipatory postural adjustments and spatial organization of motor cortex: evidence of adaptive compensations in healthy older adults." Journal of Neurophysiology **120**(6): 2796-2805.

Sparing, R., D. Buelte, I. G. Meister, T. Pauš and G. R. Fink (2008). "Transcranial magnetic stimulation and the challenge of coil placement: a comparison of conventional and stereotaxic neuronavigational strategies." Human brain mapping **29**(1): 82-96.

Stephani, C., W. Paulus and M. Sommer (2016). "The effect of current flow direction on motor hot spot allocation by transcranial magnetic stimulation." Physiological Reports **4**(1).

Suzuki, M., H. Kirimoto, H. Onishi, S. Yamada, H. Tamaki, A. Maruyama and J.-i. Yamamoto (2012). "Reciprocal changes in input–output curves of motor evoked potentials while learning motor skills." Brain research **1473**: 114-123.

Svensson, P., A. Romaniello, L. Arendt-Nielsen and B. J. Sessle (2003). "Plasticity in corticomotor control of the human tongue musculature induced by tongue-task training." Experimental brain research **152**(1): 42-51.

Thickbroom, G. W., M. L. Byrnes, S. A. Archer, A. G. Kermode and F. L. Mastaglia (2005). "Corticomotor organisation and motor function in multiple sclerosis." Journal of neurology **252**(7): 765-771.

Thickbroom, G. W., M. L. Byrnes and F. L. Mastaglia (1999). "A model of the effect of MEP amplitude variation on the accuracy of TMS mapping." Clinical Neurophysiology **110**(5): 941-943.

Thickbroom, G. W., R. Sammut and F. L. Mastaglia (1998). "Magnetic stimulation mapping of motor cortex: factors contributing to map area." Electroencephalography and Clinical Neurophysiology/Electromyography and Motor Control **109**(2): 79-84.

Thompson, M., G. Thickbroom and F. Mastaglia (1997). "Corticomotor representation of the sternocleidomastoid muscle." Brain: a journal of neurology **120**(2): 245-255.

Triggs, W. J., B. Subramanium and F. Rossi (1999). "Hand preference and transcranial magnetic stimulation asymmetry of cortical motor representation." Brain research **835**(2): 324-329.

Tsao, H., L. Danneels and P. W. Hodges (2011). "Individual fascicles of the paraspinal muscles are activated by discrete cortical networks in humans." Clinical neurophysiology **122**(8): 1580-1587.

Tsao, H., M. Galea and P. Hodges (2008). "Reorganization of the motor cortex is associated with postural control deficits in recurrent low back pain." Brain **131**(8): 2161-2171.

Turnbull, G. K., S. Hamdy, Q. Aziz, K. D. Singh and D. G. Thompson (1999). "The cortical topography of human anorectal musculature." Gastroenterology **117**(1): 32-39.

Uy, J., M. C. Ridding and T. S. Miles (2002). "Stability of maps of human motor cortex made with transcranial magnetic stimulation." Brain topography **14**(4): 293-297.

van de Ruit, M. and M. J. Grey (2017). "The TMS Motor Map Does Not Change Following a Single Session of Mirror Training Either with Or without Motor Imagery." Frontiers in human neuroscience **11**: 601-601.

van de Ruit, M. and M. J. Grey (2019). "Interindividual Variability in Use-Dependent Plasticity Following Visuomotor Learning: The Effect of Handedness and Muscle Trained." J Mot Behav **51**(2): 171-184.

Van De Ruit, M., M. J. L. Perenboom and M. J. Grey (2015). "TMS brain mapping in less than two minutes." Brain Stimulation **8**(2): 231-239.

Ward, S., A. L. Bryant, B. Pietrosimone, K. L. Bennell, R. Clark and A. J. Pearce (2016). "Cortical motor representation of the rectus femoris does not differ between the left and right hemisphere." J Electromyogr Kinesiol **28**: 46-52.

Wassermann, E. M., B. Wang, T. A. Zeffiro, N. Sadato, A. Pascual-Leone, C. Toro and M. Hallett (1996). "Locating the motor cortex on the MRI with transcranial magnetic stimulation and PET." Neuroimage **3**(1): 1-9.

Weiss, C., C. Nettekoven, A. K. Rehme, V. Neuschmelting, A. Eisenbeis, R. Goldbrunner and C. Grefkes (2013). "Mapping the hand, foot and face representations in the primary motor cortex - Retest reliability of neuronavigated TMS versus functional MRI." NeuroImage **66**: 531-542.

Wilson, S., G. Thickbroom and F. Mastaglia (1995). "Comparison of the magnetically mapped corticomotor representation of a muscle at rest and during low-level voluntary contraction." Electroencephalography and Clinical Neurophysiology/Electromyography and Motor Control **97**(5): 246-250.

Wilson, S. A., G. W. Thickbroom and F. L. Mastaglia (1993). "Transcranial magnetic stimulation mapping of the motor cortex in normal subjects. The representation of two intrinsic hand muscles." Journal of the Neurological Sciences **118**(2): 134-144.

Wolf, S. L., A. J. Butler, G. I. Campana, T. A. Parris, D. M. Struys, S. R. Weinstein and P. Weiss (2004). "Intra-subject reliability of parameters contributing to maps generated by transcranial magnetic stimulation in able-bodied adults." Clinical Neurophysiology **115**(8): 1740-1747.

Zanette, G., M. Tinazzi, C. Bonato, A. Di Summa, P. Manganotti, A. Polo and A. Fiaschi (1997). "Reversible changes of motor cortical outputs following immobilization of the upper limb." Electroencephalography and Clinical Neurophysiology - Electromyography and Motor Control **105**(4): 269-279.

Zdunczyk, A., R. Fleischmann, J. Schulz, P. Vajkoczy and T. Picht (2013). "The reliability of topographic measurements from navigated transcranial magnetic stimulation in healthy volunteers and tumor patients." Acta Neurochirurgica **155**(7): 1309-1317.

Ziemann, U., G. F. Wittenberg and L. G. Cohen (2002). "Stimulation-Induced Within-Representation and Across-Representation Plasticity in Human Motor Cortex." The Journal of Neuroscience **22**(13): 5563.
