# Supplementary material for: TMS Motor Mapping Methodology and Reliability: A Structured Review
Source: Front Neurosci. 2021 Aug 19;15:709368. doi: 10.3389/fnins.2021.709368 (PMC8417420; doi:10.3389/fnins.2021.709368)
Supplement: Supplementary file 2 [file Table_2.docx]

| **Supplementary Table 2: Reported intraclass correlation coefficients for Mapping Outcome Measures by Muscle** | | | | | | |
| --- | --- | --- | --- | --- | --- | --- |
| **Muscle** | **Area** | **Volume** | **CoGx** | **CoGy** | **HSx** | **HSy** |
| Abductor digiti minimi (ADM) | 0.85 (1) ^1,c,/^ | 0.89 (1) ^1,c,/^ |  |  |  |  |
| Abductor hallucis(AH) |  |  | 0.10 (2) ^2,+^  0.64 (3) ^2,+^ | 0.89-0.93 (2) ^2, +^  0.82-0.88 (3) ^2, +^ | 0.10-0.46 (2) ^2, +^  0.16-0.20 (3) ^2, +^ | 0.89-0.93 (2) ^2, +^  0.74-0.84 (3) ^2, +^ |
| Abductor pollicis brevis (APB) | 0.68 (4) ^1,c,/^  0.34 (5) ^2,f,+^  0.63 (1) ^1,c,/^  0.91 (6) ^1,c,/^  0.60-0.93 (7) ^2,c^ | 0.89 (1) ^1,c,/^  0.90 (6) ^1,c,/^  0.75-0.94 (7) ^2,c^ | 0.30-0.45 (2) ^2,+^  0.83 (4) ^1,c,/^  0.99 (5) ^2,f, +^  0.36-0.46 (3) ^2, +^  0.80-0.90 (7) ^2,c^ | 0.72-0.83 (2) ^2,+^  0.37 (4) ^1,c,/^  0.99 (5) ^2,f, +^  0.95-0.96 (7) ^2,c^  0.70-0.76 (3) ^2, +^ | 0.1-0.52 (2) ^2,+^  0.96 (5) ^2,f, +^  0.81-0.89 (3) ^2, +^ | 0.59-0.88 (2) ^2,+^  0.98 (5) ^2,f, +^  0.77-0.89 (3) ^2, +^ |
| Biceps brachii (BB) | 0.36 (8) ^2,f^ | 0.43 (8) ^2,f^ | 0.56 (8) ^2,f^ | 0.72 (8) ^2,f^ | 0.55 (8) ^2,f^ | 0.71 (8) ^2,f^ |
| Deltoid | 0.84 (6) ^1,c,/^ | 0.73 (6) ^1,c,/^ |  |  |  |  |
| Extensor carpi radialis (ECR) | 0.60-0.90 (7) ^2,c^ | 0.88-0.90 (7) ^2,c^ | 0.80-0.88 (7) ^2,c^ | 0.94-0.98 (7)^2,c^ |  |  |
| Extensor Digitorum Communis (EDC) | 0.86 (4) ^1,c,/^ |  | 0.38-0.77 (2) ^2,+^  0.98 (9) ^2,f,~^  0.86 (4) ^1,c,/^  0.22-0.41 (3) ^2, +^ | 0.86-0.93 (2) ^2,+^  0.97 (9) ^2,f,~^  0.70 (4) ^1,c,/^  0.79-0.81 (3) ^2, +^ | 0.42-0.70 (2) ^2,+^  0.34-0.79 (3) ^2, +^ | 0.74-0.75 (2) ^2,+^  0.65-0.78 (3) ^2, +^ |
| First dorsal interosseous  (FDI) | 0.63 (4) ^1,c,/^  0.67 (10) ^3,c^  0.13-0.69 (11) ^2,c,AR^  0.74-0.87 (12) ^2,c,^  0.71-0.91 (7) ^2,c^ | 0.36-0.58 (13) ^2,c^  0.15-0.74 (11) ^2,c,AR^  0.63-0.76 (12) ^2,c,^  0.90-0.93 (7) ^2,c^ | 0.85(4) ^1,c,/^  0.31-0.98 (2) ^2,+^  0.91(14) ^2,c,\|^  0.69-0.73 (13) ^2,c^  0.45-0.68 (11) ^2,c,AR^  0.82-0.94 (12) ^2,c,^  0.21-0.44 (3) ^2, +^  0.83-0.87 (7) ^2,c^ | 0.38 (4) ^1,c,/^  0.86-0.88 (2) ^2,+^  0.39(14)^2,c,\|^  0.76-0.81 (13) ^2,c^  0.80-0.91 (11) ^2,c, AR^  0.92 (12) ^2,c,^  0.94-0.98 (7) ^2,c^  0.81-85 (3) ^2, +^ | 0.57-0.80 (2) ^2,+^  0.14-0.57 (3) ^2, +^ | 0.65-0.80 (2) ^2,+^  0.54-0.65 (3) ^2, +^ |
| Flexor carpi radialis (FCR) | 0.85 (4) ^1,c,/^ |  | 0.69 (4) ^1,c,/^ | 0.53 (4) ^1,c,/^ |  |  |
| Lateral tongue musculature | 0.34 (5) ^2,f, +^ |  | 0.99 (5) ^2,f, +^ | 0.98 (5) ^2,f, +^ | 0.76 (5) ^2,f, +^ | 0.89 (5) ^2,f, +^ |
| Lip musculature | 0.14 (5) ^2,f, +^ |  | 0.99 (5) ^2,f, +^ | 0.99 (5) ^2,f, +^ | 0.86 (5) ^2,f, +^ | 0.89 (5) ^2,f, +^ |
| Lumbar erector spinae (LES) | 0.84(15)^2,c,A,/^ | 0.87(15) ^2,c,A,/^ | 0.94(15) ^2,c,A,/^ | 0.96(15) ^2,c,A,/^ |  |  |
| Pharynx musculature | 0.76 (16) ^1,c,/^ | 0.68 (16) ^1,c,/^ |  |  |  |  |
| Plantaris muscle (PM) | 0.39 (5) ^2,f, +^ |  | 0.979 (5) ^2,f, +^ | 0.998(5) ^2,f, +^ | 0.716 (5) ^2,f, +^ | 0.831 (5) ^2,f, +^ |
| Soleus | 0.75 – 0.91 (17) ^1,c,A,/^ |  |  |  |  |  |
| Suprahyoid | 0.91 (16) ^1,c,/^ | 0.70 (16) ^1,c,/^ |  |  |  |  |
| Tibial anterior (TA) |  |  | 0.23-0.38 (2) ^2,+^  0.35-0.66 (3) ^2, +^ | 0.87-0.93 (2) ^2,+^  0.81-0.84 (3) ^2, +^ | 0.22-0.4 (2) ^2,+^  0.11-0.73 (3) ^2, +^ | 0.71-0.9 (2) ^2,+^  0.69-0.75 (3) ^2, +^ |
| ^1^ Landmark guided navigation, ^2^ Neuronavigated, ^3^Robot assisted navigation  ^c^Coarse Grid Used, ^f^Fine Grid Used  ^A^ Mapping in the active state, or ^AR^both active and passive (otherwise at rest)  \|y-axis parallel to coil orientation at 45 degrees, x axis perpendicular to that, /coil parallel to sagittal plane, +perpendicular to central sulcus, ~variable coil orientation (otherwise coil angle at 45 degrees to sagittal plane)  ICC: High: >0.75, moderate: 0.5-0.74, low: <0.49; A range (i.e., 0.13-0.69) typically indicates the use of several methods (e.g., traditional or pseudorandom mapping) or measures from the dominant and non-dominant hand. A range was included to capture the largest extent of reliability reported. | | | | | | |

1. Mortifee P, Stewart H, Schulzer M, Eisen A. Reliability of transcranial magnetic stimulation for mapping the human motor cortex. Electroencephalography and Clinical Neurophysiology/ Evoked Potentials. 1994;93(2):131-7.

2. Forster MT, Senft C, Hattingen E, Lorei M, Seifert V, Szelenyi A. Motor cortex evaluation by ntms after surgery of central region tumors: A feasibility study. Acta Neurochirurgica. 2012;154(8):1351-9.

3. Forster MT, Limbart M, Seifert V, Senft C. Test-retest reliability of navigated transcranial magnetic stimulation of the motor cortex. Neurosurgery. 2014;10 Suppl 1:51-5; discussion 5-6.

4. Malcolm MP, Triggs WJ, Light KE, Shechtman O, Khandekar G, Gonzalez Rothi LJ. Reliability of motor cortex transcranial magnetic stimulation in four muscle representations. Clinical Neurophysiology. 2006;117(5):1037-46.

5. Weiss C, Nettekoven C, Rehme AK, Neuschmelting V, Eisenbeis A, Goldbrunner R, et al. Mapping the hand, foot and face representations in the primary motor cortex - Retest reliability of neuronavigated TMS versus functional MRI. NeuroImage. 2013;66:531-42.

6. Oliveri M, Brighina F, La Bua V, Buffa D, Aloisio A, Fierro B. Reorganization of cortical motor area in prior polio patients. Clinical Neurophysiology. 1999;110(5):806-12.

7. Cavaleri R, Schabrun SM, Chipchase LS. The reliability and validity of rapid transcranial magnetic stimulation mapping. Brain Stimulation. 2018;11(6):1291-5.

8. Sankarasubramanian V, Roelle SM, Bonnett CE, Janini D, Varnerin NM, Cunningham DA, et al. Reproducibility of transcranial magnetic stimulation metrics in the study of proximal upper limb muscles. J Electromyogr Kinesiol. 2015;25(5):754-64.

9. Kraus D, Gharabaghi A. Neuromuscular Plasticity: Disentangling Stable and Variable Motor Maps in the Human Sensorimotor Cortex. Neural Plasticity. 2016;2016.

10. Meincke J, Hewitt M, Batsikadze G, Liebetanz D. Automated TMS hotspot-hunting using a closed loop threshold-based algorithm. Neuroimage. 2016;124:509-17.

11. Ngomo S, Leonard G, Moffet H, Mercier C. Comparison of transcranial magnetic stimulation measures obtained at rest and under active conditions and their reliability. Journal of neuroscience methods. 2012;205(1):65-71.

12. Van De Ruit M, Perenboom MJL, Grey MJ. TMS brain mapping in less than two minutes. Brain Stimulation. 2015;8(2):231-9.

13. McGregor KM, Carpenter H, Kleim E, Sudhyadhom A, White KD, Butler AJ, et al. Motor map reliability and aging: A TMS/fMRI study. Experimental Brain Research. 2012;219(1):97-106.

14. Littmann AE, McHenry CL, Shields RK. Variability of motor cortical excitability using a novel mapping procedure. J Neurosci Methods. 2013;214(2):137-43.

15. Cavaleri R, Chipchase LS, Massé-Alarie H, Schabrun SM, Shraim MA, Hodges PW. Corticomotor reorganization during short-term visuomotor training in the lower back: A randomized controlled study. Brain and Behavior. 2020;10(8):e01702.

16. Plowman-Prine EK, Triggs WJ, Malcolm MP, Rosenbek JC. Reliability of transcranial magnetic stimulation for mapping swallowing musculature in the human motor cortex. Clinical Neurophysiology. 2008;119(10):2298-303.

17. Lewis GN, Signal N, Taylor D. Reliability of lower limb motor evoked potentials in stroke and healthy populations: how many responses are needed? Clinical Neurophysiology. 2014;125(4):748-54.
